# Traits Contributing to the Autistic Spectrum

**DOI:** 10.1371/journal.pone.0012633

**Published:** 2010-09-08

**Authors:** Colin D. Steer, Jean Golding, Patrick F. Bolton

**Affiliations:** 1 Social and Community Medicine, University of Bristol, Bristol, United Kingdom; 2 King's College London, Institute of Psychiatry, London, United Kingdom; VU University Medical Center and Center for Neurogenomics and Cognitive Research, VU University, Netherlands

## Abstract

**Background:**

It is increasingly recognised that traits associated with autism reflect a spectrum with no clear boundary between typical and atypical behaviour. Dimensional traits are needed to investigate the broader autism phenotype.

**Methods and Principal Findings:**

Ninety-three individual measures reflecting components of social, communication and repetitive behaviours characterising autistic spectrum disorder (ASD) were identified between the ages of 6 months and 9 years from the ALSPAC database. Using missing value imputation, data for 13,138 children were analysed. Factor analysis suggested the existence of 7 factors explaining 85% of the variance. The factors were labelled: verbal ability, language acquisition, social understanding, semantic-pragmatic skills, repetitive-stereotyped behaviour, articulation and social inhibition. Four factors (1, 3, 5 and 7) were specific to ASD being more strongly associated with this phenotype than other co-morbid conditions while other factors were more associated with learning difficulties and specific language impairment. Nevertheless, all 7 factors contributed independently to the explanation of ASD (p<0.001). Exploration of putative genetic causal factors such as variants in the *CNTNAP2* gene showed a varying pattern of associations with these traits. An alternative predictive model of ASD was derived using four individual measures: the coherence subscale of the Children's Communication Checklist (9y), the Social and Communication Disorders Checklist (91 m), repetitive behaviour (69 m) and the sociability subscale of the Emotionality Activity and Sociability measure (38 m). Although univarably these traits performed better than some factors, their combined explanations of ASD were similar (R^2^ = 0.48).

**Conclusions and Significance:**

These results support the fractional nature of ASD with different aetiological origins for these components despite pleiotropic genetic effects being observed. These traits are likely to be useful in the exploration of ASD.

## Introduction

Autism has traditionally been conceptualised as a qualitatively distinct behavioural syndrome, characterised by impairments in social interaction and communication coupled with restricted, repetitive or stereotyped patterns of behaviour, interests and activities [1], [2]. The syndrome emerges during the second year and unfolds over the next 2 years. The subtler manifestations may not become apparent until middle to late childhood. It is more commonly found in males, is associated with intellectual disability and speech/language impairments, as well as various indicators of neurodevelopmental abnormality. It usually persists into and throughout adult life.

Recent behaviour genetic studies have suggested however that the traditional model of autism as a distinct syndrome needs to be revised. Thus, twin and family data have demonstrated that the liability to autism also confers a risk for a broader range of manifestations that include other forms of pervasive developmental disorder (PDD), such as atypical autism, Asperger's syndrome and ‘other’ PDD, as well as subtler manifestations that extend beyond traditional diagnostic boundaries [3], [4]. These findings have increasingly led to the concept of an autistic spectrum disorder (ASD) with a range of manifestations. They have also raised questions about where the boundaries should be drawn between ASD and variations in ‘typical’ development in social communication and play. The lack of any clear boundary between typical and atypical behaviour has led to the suggestion that ASD represents the extreme of a normally distributed continuum [5], [6]. It is increasingly recognised, therefore, that there is a need to study dimensional as well as categorical constructs of the phenotype.

Moreover, the findings from population based twin studies have raised the possibility that rather than constituting a cohesive syndrome, ASD may instead represent a ‘compound’ phenotype that may be fractionated into different components each having separate as well as shared genetic and environmental causes [7]. At present, however, the evidence supporting the multi-dimensional model of the phenotype has been inconsistent. Various factor analytic studies have suggested up to 6 factors [8], [9] with only two studies reporting a unitary factor [10], [11]. More recent studies have reported different findings with studies supporting two or three factor models [12]–[14] and a 5 factor structure [15].

The inconsistencies amongst the findings may be attributed to various methodological issues, including differences in sampling strategies, age structure and assessment instruments.

A proper test of the contending models of the architecture of the phenotype can only be undertaken by studying population based samples and analyzing measures that cover the full range of manifestations of the putative quantitative traits. Moreover, because these traits unfold with development and become increasingly differentiated and differentiable, longitudinal data with repeat measures obtained at specific points in development has special value in that it enables examination of the developmental emergence of the phenotype as well as the identification of enduring traits rather than transient states.

Our aims in this study were twofold. First, we wished to identify putative predictors of autism and to test the uni- versus multi- dimensional models of the broader autism phenotype by analyzing data from a large, prospective cohort study – The Avon Longitudinal Study of Parents and Children (ALSPAC). This represents the first prospective longitudinal study to explore the architecture of phenotypes associated with ASD. Our approach was to undertake a factor analysis of putative traits and to validate the factors by examining their predictive validity with regard to the diagnosis of ASD, as well as the specificity of their associations to ASD compared with other psychiatric, cognitive and developmental conditions co-morbid with ASD.

Our second aim was to illustrate how the traits could be used to identify and characterize correlates of the broader autism phenotype. Within this investigation, we have focused on the genetic correlates reporting the associations with common polymorphisms in the contactin and cadherin genes. These variants have previously been reported to be associated with ASD and key components of ASD [16]–[19].

## Methods

### Ethics statement

Ethical approval for the study was obtained from the ALSPAC Law and Ethics Committee and the Southmead, Frenchay, UBHT and Weston Research Ethics Committees. Written consent was obtained from participants to allow use of anonymized linked data for research by bona fide scientists.

### The Study Sample

ALSPAC was established to explore the environmental, social, psychological and genetic factors associated with child health and development. It recruited 14,541 pregnant women in the Bristol area who had an expected delivery date between April 1991 and December 1992. From these pregnancies, 13,971 children from the study were alive at age 7 years [20]. Since the initial recruitment, 416 new children including one ASD case have participated in the study and are included in the data used in this report.

### Autistic Spectrum Disorder

Children in the ALSPAC sample with ASD were identified either from community paediatric records or from the special educational needs database for the region [21]. Clinical records were reviewed by a consultant paediatrician to confirm diagnoses according to ICD-10 criteria [2]. In particular, this review ensured that a multi-disciplinary assessment had been made. The identification and review of cases was blind to the data used in this study. There were 86 such children identified by age 11 years giving a prevalence of 62 per 10,000 children based upon the original recruited sample of 13, 971 children. The number of cases should be considered a maximum with actual numbers available for analysis depending on the response rates for other data at particular ages of interest.

The prevalence estimate is somewhat lower than other estimates. A recent study by Baron-Cohen et al has suggested a prevalence rate of 0.9% based upon a survey of special educational needs (SEN) amongst 96 schools. This estimate was revised upwards to 1.6% when maternal report of ASD status and symptoms were considered [22]. It is likely that our prevalence estimate is a lower estimate due to stricter inclusion criteria. Using similar criteria and similar sources of information to the Baron-Cohen study would have revised our prevalence estimate to 1.5% (paper in preparation).

### Identification of individual measures

The ALSPAC dataset was searched for measures relating to the main features of ASD with respect to social/communication problems and repetitive-stereotyped behaviour gathered up to age 9 years. In all, 93 traits were identified of which 46 related to 12 standard tests [23]–[34]. However, many of these measures were abbreviated, adapted or subscales modified in order to make it practicable to collect data in such a large cohort. Details of the measures selected for this study can be found in Methods S1 and Table S1.

### Co-morbid conditions

Although not considered a core requirement for the diagnosis of ASD, many children exhibit other traits such as learning difficulties, specific language impairment (SLI), ADHD, ODD/CD, anxiety problems and SEN. Learning difficulties was defined by IQ <70 as assessed at 8y by trained psychologists. SLI was derived from parental report of persistent problems with speech at 8½y. Those children with learning difficulties were excluded from this definition. ADHD, ODD/CD and anxiety problems were proxy DSM-IV diagnoses using the Development and Well-Being Assessment (DAWBA) questionnaire completed by the parents and SDQ assessments completed by the child's teacher at 7½y [23], [24]. Children with SEN were identified from the Pupil Level Annual School Census (PLASC) returns for the 2003/4 academic year. Children with short-term needs (referred to in the census as *school action*) were not considered as SEN.

### Genetic markers

DNA was extracted from blood samples taken from the children at various ages [35]. Genotyping of rs4307059 (intergenic region between *CDH9* and *CDH10* genes) and rs2710102, rs17326239 and rs7794745 (*CNTNAP2*) SNPs was undertaken by KBioscience Ltd using a competitive allele specific PCR system (KASPar) for SNP analysis. Failure rates ranged from 3.6% to 8.9% leaving data from 9126 white ethnic children available for analysis (82.8% of these having data on all 4 SNPs). The first two genetic variants were in Hardy-Weinberg equilibrium (p>0.4) but the latter two SNPs showed evidence of disequilibrium (p<0.01). Minor allele frequencies were 38.0% (C), 49.6% (A), 35.7% (G) and 30.1% (T) respectively.

### Statistical Analyses

Missing value imputation was undertaken using the method of imputation by chained equations [36]. A single imputed estimate was derived based upon the predicted values from each imputation equation using the other 92 individual measures as predictors. Imputations were repeated using different initial missing value estimates to provide assurance that a global minimum was obtained. Imputed values were constrained to lie within the feasible range of values for each measure.

Principal factor analysis of the correlation matrix was used to investigate the latent structure of factors underlying the variables. Two alternative methods of rotation, varimax and promax, were employed to simplify the pattern of loadings from this analysis. Scree plot, Parallel Analysis and goodness-of-fit statistics (see Methods S2) assisted in the choice of the number of factors [37]–[39]. Factor scores were calculated from the factor loadings rather than summing the major individual measures associated with each factor due to the lower determinacy of this latter method [40].

In order to exploit the prospective longitudinal data available and to test the notion that the architecture of the phenotype would become increasingly differentiated and differentiable as development unfolded, we conducted our factor analysis focusing on measures obtained during four different developmental epochs: 6–18 months; 18–38 months; 42–77 months and 81 months –9 years. These developmental periods were selected because of the usual developmental course of autism and because they corresponded to periods that related to some of our key trait measures.

As the individual measures were selected from a wide range of measures (general and autism specific questions and questionnaire as well as direct observational measures) that were collected at different time points in development, it was necessary to consider the possibility that the derived factors scores might not index the underlying ASD traits as well as some of the measures that were specifically developed to assess autistic traits. Accordingly, we also identified the best measures in predicting ASD using a subset regression approach assuming 3 predictors reflecting the diagnostic triad.

Additional analyses were undertaken to examine the specificity of the identified trait measures, whether factors or individual measures, to ASD. This was achieved in two parts. Firstly, logistic regression was used to establish the most important traits in predicting ASD status. Since it is important that traits predict ASD rather than male gender, these analyses were adjusted for gender [41]. In these analyses, traits were treated as linear covariates. Non-linearity was investigated using quadratic terms. Secondly, further analyses investigated whether these associations related specifically to ASD as distinct from other co-morbid conditions not considered central to the diagnosis of ASD. Linear regression analyses adjusting for gender were used to compare the prediction of the traits by such diagnoses. All traits were standardized to have a variance of one to allow comparison of the effect sizes across traits.

In addition, the pattern of associations between identified traits and genetic correlates of ASD was examined to determine whether there was any evidence to suggest different aetiological origins or modifying influences on individual traits. If different genes are associated with the traits, this would support different aetiological causes or at least strong associations with other traits having a causal link. On the other hand, if the associations were restricted to a single gene, this might be interpreted as the traits reflecting different manifestations of a single underlying cause. These analyses were restricted to those children of white ethnic origin. Minor allele frequencies can vary by ethnic background and although it is possible to adjust for this feature, the complication of mixed race backgrounds makes it simpler to restrict the data used in such analyses.

A list of abbreviations used in this paper is provided in Methods S3.

## Results

### Sample characteristics

Basic descriptive data of the individual measures used in these analyses and differences between observed and imputed data are reported in Table S2. Data on at least one individual measure were available for 13,138 children (91.3%) with complete data on 2481 children (17.2%). There were 80 ASD cases identified within this sample. Missing data represented 30% of all data items but was slightly less prevalent amongst the ASD cases (26%). However, this difference was compatible with random variation (p = 0.220). Of the 9375 children with observed data on 47 or more of the individual measures, 11% of the data items were missing. Sample attrition ranged from 14% to 48%. An indication of the predictive ability of the imputation equations is given in Table S3. The estimated maximum communality is the R^2^ of one individual measure on the remaining 92 measures or in other words the imputation equation.

The 80 ASD cases represented 28 Childhood autism, 14 Atypical, 21 Asperger's syndrome, 3 other or unspecified pervasive developmental disorders and 14 with an unknown ICD-10 classification identified from educational records.

About 99% of children were consistently reported to use English as their main language based upon PLASC (9–11y censuses) and parental reports between the ages of 38 m and 8y. This included all of the ASD cases. Only 65 children consistently reported some other main language with 96 children having inconsistent responses. This latter group included those who increasingly used English as they became older.

In all, 5.1% of children were classified as non-white. This percentage did not vary by ASD status.

### Factor analysis

**Figure 1:**
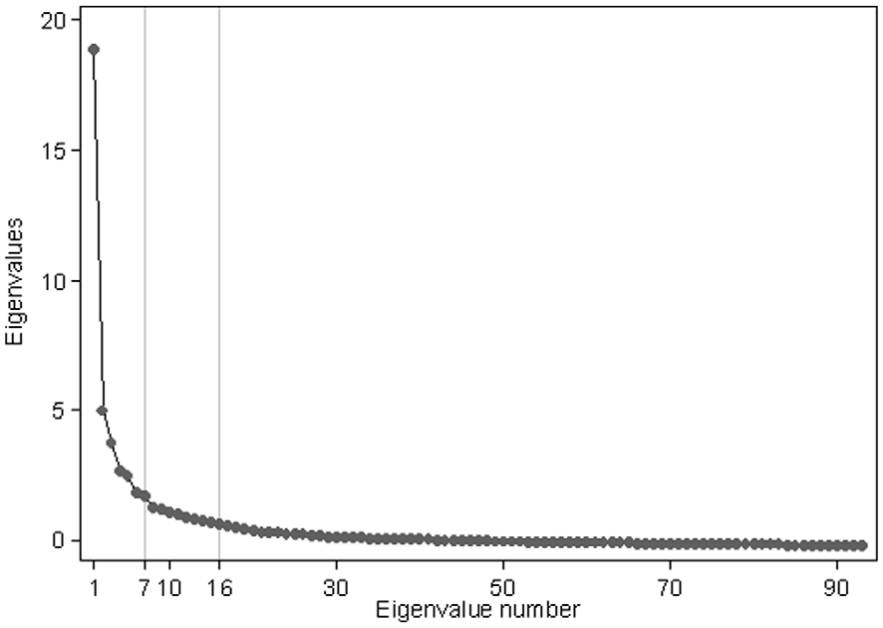
Scree plot of eigenvalues from a factor analysis of the correlation matrix for 93 traits (N = 13,138). Seven factors were retained based upon changes in the slope associated with the eigenvalues. Parallel analysis and CFI criteria suggested 16 factors.

Analysis of all the observed and imputed values showed a first factor explaining 44% of the variance (Figure 1). This scree plot suggested two points of inflection occurring after 3 and 7 factors explaining 65% and 85% of the variance. SRMR and RMSEA statistics suggested similar solutions although 4 or 9 factors respectively were required to achieve the criterion of <0.05 for a good fit (see Table 1). In contrast, Parallel Analysis suggested a larger number of factors. Using 1000 random permutations of the data, observed eigenvalues exceeded the 95^th^ centile of this null distribution up to the 16^th^ factor with a critical eigenvalue of 0.556 and 104% variance explained. This solution was also supported by the CFI. To achieve a balance between parsimony and variance explained, a 7-factor solution was chosen.

**Table 1:** Goodness-of-fit tests for models retaining 1 to 20 factors from an analysis of 93 traits (N = 13,138).

Number of Factors	Residual df	Chi-square related tests			Residual correlations			Variance explained
		χ^2^	RMSEA	CFI	SRMR	>0.1	>0.05	
Null	4278	602645	0.103	0.000	0.262	73.7	93.9	0.0
1	4185	347777	0.079	0.426	0.084	12.7	37.0	44.2
2	4093	278850	0.071	0.541	0.067	7.0	26.7	55.8
3	4002	230499	0.066	0.621	0.055	4.5	18.4	64.5
4	3912	198199	0.061	0.675	0.048	2.8	14.7	70.7
5	3823	172549	0.058	0.718	0.041	2.2	10.1	76.5
6	3735	155107	0.056	0.747	0.037	1.9	8.6	80.7
7	3648	139399	0.053	0.773	0.033	1.8	6.5	84.7
8	3562	124820	0.051	0.797	0.030	1.5	6.0	87.6
9	3477	111664	0.049	0.819	0.028	1.2	4.9	90.3
10	3393	101096	0.047	0.837	0.026	1.1	4.1	92.7
11	3310	89025	0.044	0.857	0.024	0.9	3.6	95.1
12	3228	82315	0.043	0.868	0.022	0.7	3.0	97.0
13	3147	76152	0.042	0.878	0.020	0.6	2.3	98.9
14	3067	71111	0.041	0.886	0.018	0.4	2.0	100.7
15	2988	67511	0.041	0.892	0.017	0.2	1.7	102.3
16	2910	64403	0.040	0.897	0.016	0.2	1.4	103.6
17	2833	62037	0.040	0.901	0.014	0.2	1.3	104.9
18	2757	58874	0.039	0.906	0.013	0.1	1.1	106.0
19	2682	56837	0.039	0.909	0.012	0.1	0.9	107.0
20	2608	55531	0.039	0.912	0.012	0.1	0.7	107.8

CFI = Comparative Fit index.

RMSEA = Root mean square error of approximation.

SRMR = Standardised root mean square residual.

All the model χ^2^ values were highly significant (p<0.0001). For the 7 factors in this study, the overall fit statistics suggested a reasonable to good fit. Examination of the 66 residual correlations >0.1 showed that they clustered within the factor structure: 6 related to Factor 2 variables 6–24 m (6.6% of all inter-correlations within this group), 7 (19.4%) related to Factors 1/2 variables 30–42 m, 8 (7.6%) related to Factor 1 variables 57 m–9y, 4 (8.9%) to Factor 3 variables, 10 (12.8%) to Factor 4, 7 (19.4%) to Factor 5, 12 (21.8%) to Factor 6 and 12 (18.2%) to Factor 7.

The variance explained is expressed as a percentage of the sum of the communalities. This latter measure can exceed 100% due to the presence of negative eigenvalues. Residual correlations >0.1 and >0.05 are reported as a percentage of the 4278 pairwise correlations.

The results from varimax rotation are shown in Table 2. An arbitrary loading of 0.3 was chosen to identify the major factors associated with each individual measure and to assist in the interpretation of factors. In all, 65 measures loaded on only one factor with 10 failing to reach this critical value on any of the 7 factors. While these ten measures might have suggested the presence of additional factors, their low communalities was perhaps more indicative of considerable measurement error or other sources of uniqueness in these variables (see Table S3). Using oblique instead of orthogonal rotation did not substantially change the factor structure (see Table S4). With the correlations between these oblique factors ranging from −0.088 to 0.541 and the general similarity in factor structure, it was decided to retain the orthogonal factors. These factors were interpreted as:

Factor 1: Verbal ability

Factor 2: Language acquisition

Factor 3: Social understanding

Factor 4: Semantic-pragmatic skills

Factor 5: Repetitive-stereotyped behaviour

Factor 6: Articulation

Factor 7: Social inhibition

**Table 2:** Factor analysis of 93 traits after varimax rotation (N = 13,138).

Age	Trait	Factor Loadings						
		1	2	3	4	5	6	7
6 m	DDST – Communication	0.02	**0.35**	0.05	−0.09	−0.01	0.05	0.03
	Pretend play	0.00	0.27	0.06	−0.10	−0.01	0.05	0.01
15 m	CDI – understand score	0.08	**0.60**	0.16	−0.02	0.09	0.04	0.05
	CDI – Vocabulary	0.03	**0.78**	0.06	0.03	0.05	0.07	0.01
	CDI – response to language	0.10	0.16	0.16	0.05	0.08	0.01	0.05
	CDI – imitates words	0.09	**0.56**	0.03	0.08	0.02	0.09	0.08
	CDI – gestures	0.11	**0.55**	0.22	−0.06	0.09	0.01	0.11
	CDI – objects	0.09	**0.63**	0.19	0.06	0.09	−0.02	0.04
18 m	DDST – communication	0.13	**0.75**	0.05	0.09	0.02	0.16	0.06
	Pretend play	0.15	**0.35**	0.16	−0.04	0.09	0.00	0.07
24 m	CDI – Vocabulary	0.20	**0.79**	0.00	**0.31**	−0.02	0.15	0.01
	CDI – grammar (regular)	0.13	**0.69**	−0.02	0.26	−0.05	0.21	0.04
	CDI – grammar (irregular)	0.09	**0.75**	0.00	0.18	−0.03	0.13	−0.01
	CDI – combines words	0.22	**0.56**	−0.03	**0.34**	−0.04	0.16	0.06
30 m	Pretend play	0.13	**0.47**	0.25	0.02	0.04	−0.06	0.09
38 m	CDI – Vocabulary	**0.58**	**0.37**	0.08	0.24	0.05	0.14	0.06
	CDI – grammar (regular)	**0.47**	**0.39**	0.06	**0.34**	−0.03	0.21	0.07
	CDI – grammar (irregular)	**0.40**	**0.44**	0.06	0.22	−0.02	0.14	0.04
	CDI – complexity	**0.49**	**0.30**	0.04	**0.34**	−0.02	0.14	0.08
	CDI – combines words	**0.70**	0.16	0.04	0.18	0.07	0.11	0.11
	Communication	**0.43**	0.29	0.10	**0.39**	0.08	0.24	0.12
	Intelligibility	**0.46**	0.22	0.06	0.24	0.03	0.28	0.09
42 m	Pretend play	0.22	**0.41**	0.32	0.01	0.07	−0.07	0.12
57 m	Communication	**0.76**	0.14	0.18	0.14	0.22	0.17	0.11
	Musical	**0.47**	0.26	0.22	0.12	0.05	0.07	0.09
	Intelligibility	0.25	0.08	0.05	0.04	0.04	0.24	0.07
	Combines words	**0.72**	0.07	0.08	0.12	0.16	0.05	0.13
69 m	Communication	**0.75**	0.09	0.20	0.12	0.29	0.14	0.09
	Musical	**0.49**	0.21	0.23	0.09	0.14	0.04	0.09
	Intelligibility	0.20	0.03	0.07	0.04	0.06	0.17	0.06
	Combines words	**0.73**	0.05	0.09	0.06	0.19	0.03	0.11
81 m	Communication	**0.73**	0.07	0.22	0.13	0.32	0.14	0.11
	Musical	**0.45**	0.17	0.27	0.11	0.16	0.08	0.09
	Intelligibility	0.21	0.05	0.06	0.04	0.07	0.15	0.05
	Combines words	**0.66**	0.00	0.09	0.01	0.20	0.01	0.09
9y	CCC – intelligibility & fluency	**0.59**	0.12	0.11	0.15	0.15	**0.38**	0.15
	CCC – syntax score	**0.69**	0.05	0.14	0.19	0.24	0.15	0.11
	CCC – coherence	**0.49**	0.14	**0.30**	**0.30**	0.27	0.28	0.15
42 m	Rutter Prosocial	0.14	0.28	**0.58**	0.02	0.03	0.03	0.16
47 m	SDQ Prosocial	0.12	0.23	**0.60**	0.05	0.00	0.04	0.14
57 m	Empathy	0.21	0.13	**0.58**	0.13	0.15	0.08	0.04
69 m	Empathy	0.22	0.09	**0.59**	0.17	0.20	0.08	0.01
81 m	Empathy	0.20	0.09	**0.62**	0.15	0.16	0.11	0.01
	SDQ Prosocial	0.06	0.16	**0.74**	0.02	0.07	0.07	0.10
91 m	SCDC	0.17	0.01	**0.54**	0.27	**0.32**	0.17	−0.02
97 m	SDQ Prosocial	0.05	0.14	**0.74**	0.03	0.08	0.05	0.10
9y	SDQ Prosocial	0.05	0.15	**0.71**	−0.03	0.08	0.06	0.11
	CCC – conversational rapport	0.25	0.12	**0.43**	0.25	0.26	0.13	0.31
38 m	Echoes what said	−0.03	0.13	−0.01	**0.43**	0.03	0.09	0.02
57 m	Echoes what said	0.07	0.00	0.05	**0.52**	0.10	0.09	0.07
69 m	Echoes what said	0.14	−0.02	0.07	**0.52**	0.14	0.08	0.08
81 m	Echoes what said	0.14	−0.05	0.09	**0.48**	0.15	0.07	0.10
	Nonverbal communication	0.16	0.05	0.21	0.29	0.11	0.05	0.10
8y	WOLD – comprehension	0.24	0.09	0.03	**0.40**	0.02	−0.07	0.01
	WOLD – oral expression	**0.38**	0.18	0.02	**0.50**	0.02	−0.01	0.01
	Nonword repetition	**0.36**	0.24	−0.07	**0.40**	0.00	0.14	−0.01
	WISC – verbal IQ	**0.40**	0.11	0.01	**0.58**	0.03	−0.05	0.05
	DANVA – faces	0.21	0.15	0.13	0.18	0.08	0.01	0.07
9y	CCC – inappropriate initiation	0.06	−0.06	0.15	**0.46**	0.23	0.10	−0.26
	CCC – stereotyped conversation	0.05	−0.10	0.19	**0.51**	0.28	0.18	−0.02
	CCC – conversational context	0.29	0.01	**0.31**	**0.55**	**0.31**	0.14	0.06
18 m	Repetitive behaviour	0.03	0.06	0.07	0.15	**0.31**	0.08	0.05
30 m	Repetitive behaviour	0.08	0.06	0.03	0.09	**0.44**	0.04	0.00
42 m	Repetitive behaviour	0.12	0.04	0.06	0.03	**0.49**	0.04	0.04
57 m	Repetitive behaviour	0.12	0.04	0.09	0.05	**0.56**	0.07	0.06
69 m	Repetitive behaviour	0.15	0.03	0.09	0.08	**0.58**	0.08	0.05
77 m	Repetitive behaviour	0.13	0.01	0.10	0.04	**0.55**	0.09	0.05
91 m	DAWBA – Number compulsions	0.19	−0.04	0.10	0.19	**0.65**	−0.01	0.13
	DAWBA – Compulsions score	0.22	−0.04	0.11	0.18	**0.69**	−0.02	0.12
	DAWBA – Tics or twitches	0.08	0.03	0.07	0.02	0.27	0.06	0.00
38 m	Stumbles on words	−0.12	0.03	0.05	0.12	0.05	**0.30**	0.07
	Prefers gestures	0.19	0.20	0.03	0.28	0.01	**0.30**	0.12
57 m	Stumbles on words	0.05	0.06	0.09	0.26	0.10	**0.41**	0.11
	Prefers gestures	0.15	0.11	0.07	0.29	0.05	**0.37**	0.18
	Pronouncing certain sounds	0.14	0.15	0.02	−0.08	0.04	**0.54**	0.01
69 m	Stumbles on words	0.11	0.06	0.10	0.27	0.13	**0.44**	0.09
	Prefers gestures	0.18	0.11	0.08	0.27	0.07	**0.38**	0.16
	Pronouncing certain sounds	0.19	0.12	0.02	−0.07	0.06	**0.59**	0.01
81 m	Stumbles on words	0.18	0.03	0.10	0.25	0.09	**0.42**	0.11
	Prefers gestures	0.20	0.09	0.08	0.25	0.08	**0.35**	0.18
	Pronouncing certain sounds	0.24	0.12	0.04	−0.07	0.09	**0.54**	0.01
38 m	EAS – Sociability	0.06	0.10	0.04	0.04	−0.02	−0.05	**0.53**
	Stays mainly silent	**0.31**	0.13	0.04	0.06	0.06	0.13	**0.35**
	Avoids eye contact	0.13	0.03	0.11	0.18	0.07	0.10	**0.29**
57 m	EAS – Sociability	0.06	0.08	0.02	0.03	−0.02	−0.06	**0.61**
	Stays mainly silent	0.14	0.08	0.02	−0.04	0.05	0.12	**0.52**
	Avoids eye contact	0.05	−0.01	0.18	0.17	0.08	0.10	**0.39**
69 m	EAS – Sociability	0.04	0.08	0.05	−0.01	−0.01	−0.04	**0.60**
	Stays mainly silent	0.12	0.05	0.01	−0.06	0.08	0.13	**0.54**
	Avoids eye contact	0.05	−0.02	0.21	0.17	0.10	0.14	**0.40**
81 m	Stays mainly silent	0.07	0.05	0.03	−0.02	0.06	0.13	**0.51**
	Avoids eye contact	0.07	0.01	0.25	0.16	0.11	0.16	**0.36**
91 m	DAWBA – Social fears	0.12	−0.02	0.16	0.15	0.11	0.12	0.25
	Variance explained	20.5%	16.0%	12.3%	11.6%	8.9%	7.8%	7.7%

See Methods S3 for definitions of the abbreviations associated with the individual measures. Loadings ≥0.3 are shown in bold. This criterion is used to aid interpretation rather than imply any significant deviation from zero. The total variance explained by the 7 factors was 84.7%. The first 7 domains match the retained factors: 1 *Verbal ability*, 2 *Language acquisition*, 3 *Social understanding*, 4 *Semantic-pragmatic skills*, 5 *Repetitive Behaviour*, 6 *Articulation* and 7 *Social inhibition*.

Examination of the correlation residuals showed that these factors satisfactorily explained the correlations between variables associated with different factors with the main deviations existing within the same factor (see Table 1). This would seem to imply that more minor factors, if they exist, form a hierarchical structure splintering the 7 main factors. Figure S1 illustrates how 4 major factors might be separated into 10 minor factors.

All factor scores had high determinacy (range 0.89 to 0.96).

### Sensitivity of the factor structure to data characteristics

There were a number of features associated with the data used in this study which may have impacted on the factor structure. These included the imputation process, the use of a population-based sample and the inclusion of repeat measures at different ages. It is perhaps not surprising that, as one reduces the amount of information in the data set, greater discrepancies with the above results emerge. Hence, reducing the sample size by using observed pairwise correlations and then completely observed data led to increasing discrepancies in the factor structure compared to the imputed data set. But the discrepancies were minor reflecting about 3% of the loadings. It is perhaps to be expected that imputation had little impact on the factor structure. Where the imputation was less precise, this led to a low maximum communality or R^2^. As a consequence, the associated individual measures tended to have a more minor role in the factor structure and in most cases failed to load highly on any factor.

More discrepancies in the factor structure were noted when particular subgroups of the population were analysed and hence further reductions in sample size. But the most severe discrepancies were noted when the data were restricted in terms of variables rather than observations. Nevertheless, even in this case when repeat measures were excluded reducing the variable list to 44 individual measures, 87% of the factor loadings were equivalent (see Results S1, Table S4).

### Stability of the factor structure across time

As children became older, the factor structure became more elaborate with an increasing numbers of factors: one, five, six and seven factors in the periods 6–15 m, 18–38 m, 42–77 m and 81 m–9y respectively (see Table S5, Figure S2). To some extent, these results may have reflected the availability of data and the ability to assess children more intensely at older ages. But in addition, they may also have reflected different developmental trajectories with differences between children becoming more extreme with age. Most individual measures loaded highly on their expected factor. The exceptions to this general pattern were *Stumbles on words* and *Prefers gestures* (at 57 m and 69 m) which were more associated with Factor 3 (*Social understanding*) than Factor 6 (*Articulation*). In addition, the 8y measures were identified as a separate factor rather than associated with Factor 4 (*Semantic-pragmatic skills*). This feature was to some extent mirrored in the overall analyses of 93 measures if 8 instead of 7 factors were retained or in the analysis of this factor's individual measures (see Figure S1). The factor scores derived at different ages correlated in the expected manner (see Table 3). Overall, these results support the 7 major factors although, as previously noted, other more minor factors may exist.

**Table 3:** Correlations between factors generated from variables for different age ranges and for all ages (N = 13,138).

Factor	6–15 m		18–38 m			42–77 m		81 m–9y
	18–38 m	All	42–77 m	81 m–9y	All	81 m–9y	All	All
1			0.58	0.43	0.70	0.77	0.93	0.83
2	0.67	0.86			0.90			
3						0.68	0.82	0.91
4			0.35	0.31	0.47	0.46	0.67	0.87
5			0.42	0.13	0.49	0.27	0.79	0.69
6			0.20	0.21	0.37	0.54	0.81	0.72
7			0.41	0.34	0.53	0.45	0.92	0.66

Factors derived from analysis of all ages: 1 *Verbal ability*, 2 *Language acquisition*, 3 *Social understanding*, 4 *Semantic-pragmatic skills*, 5 *Repetitive Behaviour*, 6 *Articulation* and 7 *Social inhibition*. Not all factors were identified for each age range. Hence, for example, the comparisons for the 6–15 m age range only show one factor and are only compared with the equivalent factor derived for 18–38 m and for all ages. This factor was not identified for age ranges 42–77 m and 81 m–9y.

At age 18–38 m, *Echoes what said 38 m* failed to load highly on any of the 5 factors identified for this age range but was included as a 6^th^ ‘factor’ and used as a proxy for Factor 4.

At age 81 m–9y, an estimate of Factor 4 was calculated by summing two factors (2^nd^ and 3^rd^ factors, see Table S5 part D) derived from the factor analysis for this age range.

All correlations were highly significant (p<0.0001).

### Factor mean score

Although factor scores were nominally orthogonal, this overall relationship masked associations at the extremes. So for example, the correlations between factor scores in the bottom quartile of Factor 1 ranged between −0.04 and 0.32 (average 0.12). This apparent co-morbidity in many ways mimics the multi-factorial nature of ASD itself and raises the possibility that a combined factor score may provide further insights not apparent or only discernible at a lower level of power in individual factors. While it is clearly possible to define a linear or non-linear combination of the factors which maximises the prediction of any outcome of interest, using a simple arithmetic average is a neutral approach which does not pre-suppose any particular outcome.

### Prediction of ASD

Table S6 summarizes the association between the worst decile on factor and individual item scores, according to the presence of a diagnosis of ASD. The predictive powers of the scores are ranked in the table considering the traits as dimensional variables. Most traits were associated with ASD diagnosis, with the prevalence of children in the worst decile for each trait, as expected, being higher for those with positive status (sensitivity) compared to those with negative status (1 – specificity).

Imputation increased sensitivity on average from 48% to 53% for ASD status. Effect sizes (log OR) in ASD analyses for individual measures were 12% higher for data with imputation compared to observed data only although standard errors were 7% higher compared to those expected from the increased sample size.

The ranking of factors 1, 3 and 7 in terms of their associations with ASD reflected the average rank of the individual measures loading highly on each factor. So for instance, the 10 individual measures associated with Factor 3 had an average rank of 13.2 while this factor itself had a rank of 15. In contrast, Factor 5 performed better with a rank of 25 compared to 57.2 for the individual measures. Inevitably, this implied that several individual measures predicted ASD status better than their associated factor. A notable example of this was Factor 2. This factor was not univariably associated with ASD status performing worse than all the individual measures associated with this factor. Similar but less extreme results were observed for Factors 4 and 6 where 90% of individual measures performed better than their associated factor. In contrast, by exploiting their orthogonal nature, a mean score for the 7 factors had the strongest univariable association with ASD status.

As noted above, some individual measures had very strong associations with ASD, in particular, various subscales of the Children's Communication Checklist (CCC) at 9y, *coherence*, *conversational context* and *conversational rapport* (ranks 2, 3 and 4 respectively), and Social and Communication Disorders Checklist (SCDC) at 91 m (rank 6) [32], [34]. These measures reflected the communication and social domains of the diagnostic triad and were to some extent specifically designed to assess ASD. Measures of repetitive-stereotyped behaviour were less predictive but two of the best measures were *DAWBA – compulsions score 91 m* and *Repetitive behaviour 69 m* (ranks 23 and 40 respectively). Some traits, *CCC – coherence 9y*, the sociability subscale of the Emotionality Activity and Sociability (EAS) measure at 38 m and 69 m, and *Stays mainly silent 69 m* enhanced the explanation of ASD even in the presence of the seven factors (p<0.011). It is interesting to note that the latter three traits were not individually strong predictors of ASD (ranks 55, 28 and 84 respectively). However, these results may indicate that they could play a more major role in multivariable models capturing variation not present in other traits.

### Multivariable associations with ASD status

The importance of particular combinations of traits in predicting ASD status was investigated using logistic regression (see Table 4). Using all available data including imputed values, each factor had a strong independent association with ASD status. Restricting the data to where at least half of the individual measures were observed did not substantially change the results in terms of effect sizes. Even using complete data with no imputation, only factors 2 and 3 showed appreciable attenuation although the impact on statistical significance was more extreme for all factors.

**Table 4:** Sensitivity analysis of the associations between ASD status and the 7 Factors or the 4 individual measures.

Trait	All Data				At least half observed				Complete data			
	OR	95% CI		p	OR	95% CI		p	OR	95% CI		p
1: Verbal ability	1.38	1.26	1.51	<0.001	1.48	1.34	1.65	<0.001	0.92	0.43	2.00	0.837
2: Language acquisition	1.65	1.26	2.17	<0.001	1.78	1.28	2.49	0.001	0.59	0.19	1.78	0.345
3: Social understanding	2.27	1.88	2.73	<0.001	2.41	1.93	3.00	<0.001	2.85	1.39	5.86	0.004
4: Semantic-pragmatic skills	1.88	1.55	2.28	<0.001	1.95	1.57	2.43	<0.001	2.11	0.77	5.74	0.144
5: Repetitive-stereotyped	1.32	1.20	1.45	<0.001	1.32	1.18	1.47	<0.001	1.42	0.87	2.31	0.159
6: Articulation	1.41	1.16	1.70	<0.001	1.58	1.27	1.95	<0.001	2.12	0.94	4.78	0.072
7: Social inhibition	1.67	1.36	2.06	<0.001	1.61	1.28	2.02	<0.001	1.32	0.68	2.55	0.410
N	13138				9375				2481			
ASD cases	80				61				4			
Increase in R^2^	0.450				0.501				0.399			
CCC – coherence 9y	1.86	1.63	2.14	<0.001	1.94	1.67	2.25	<0.001	2.18	1.81	2.63	<0.001
SCDC 91 m	1.58	1.34	1.87	<0.001	1.58	1.32	1.89	<0.001	1.62	1.31	2.01	<0.001
Repetitive behaviour 69 m	1.16	1.04	1.30	0.011	1.15	1.02	1.30	0.026	1.09	0.91	1.29	0.345
EAS – sociability 38 m	1.77	1.42	2.21	<0.001	1.79	1.41	2.29	<0.001	1.61	1.19	2.18	0.002
N	11411				9422				6055			
ASD cases	73				63				44			
Increase in R^2^	0.482				0.527				0.544			

See Methods S3 for definitions of the abbreviations associated with the individual measures. Logistic regression was used to test the association between the traits and ASD status adjusting for gender. Traits were included as linear covariates. ORs are for one SD decrease in trait score. *All data* consists of at least one individual measure with observed data (from the 93 measures used to derive the factors or from the 4 measures included in the individual measure model) with missing data being imputed. *Complete data* consists of observed data only. R^2^ are reported as the increase in the explanation of the log-likelihood compared to a model involving gender only. Gender explained about 5% of the log likelihood in all models. The estimated relative contribution of each trait using *All data* were: 19%, 5%, 31%, 17%, 13%, 5% and 10% (Factors); and, 57%, 19%, 4% and 19% (individual measures). The factor model performed similarly to the individual measure model when restricted to the same sample (increase in R^2^  = 0.493, N = 11411).

Subset regression was used to identify which individual measures combined optimally in predicting ASD (see Table S7). One of the best models reflecting the diagnostic triad involved individual measures identified in the previous section with strong univariable associations in their respective domains viz. *CCC – coherence 9y*, *SCDC 91 m* and *Repetitive behaviour 69 m*. These analyses also suggested that the contribution of the social domain could be improved by including a second measure. As a consequence, *EAS – sociability 38 m* was included as a fourth trait in the individual measure model. This model performed similarly to the factor model. While this was achieved with fewer degrees of freedom, it was to some extent data driven which may have inflated the explanation. As with the factors, the impact of imputed data on the results was generally small with the largest differences occurring when restricting to observed data only.

There was evidence of non-linear associations with ASD for *CCC – coherence 9y* (p<0.001), *SCDC 91 m* (p = 0.006) and possibly for Factor 1 (p = 0.054) but not for other traits present in Table 4. Introducing quadratic terms for these traits increased R^2^ by 0.038, 0.008 and 0.004 respectively. The ORs for other traits in these models were changed by −13% to +11%.

Combined analyses of the identified traits showed that only Factors 5 and 6 and *SCDC 91 m* failed to have an independent association. This result is surprising and indicates that there is limited overlap between the two sets of traits. However caution should be exercised with this result since this combined model may be over-defined with only 6 ASD cases per model parameter.

### Validation of the identified traits

Many of the individual measures relied on parental report which could be susceptible to potential sources of mis-reporting such as over-reporting post diagnosis and under-reporting for the first child of the family. At age 8y, 7487 of the sample attended a clinic where trained staff assessed the children. The *Wariness* subscale of the Dunedin Temperament Scale [42] was particularly associated with Factor 7 (*Social inhibition*) and *EAS – sociability 38 m* (p<0.001). An assessment of verbal fluency was associated with Factor 6 (*Articulation*) and *CCC – coherence 9y* (p<0.001).

### Specificity of the identified traits for ASD

The associations of the selected traits with ASD and 6 other co-morbid conditions are shown in Table 5. It can be seen that these conditions are much more prevalent in the ASD cases than in the general population. In particular, all but one ASD child had SEN. While for all of the individual measures the strongest negative effect was associated with ASD, the factors showed a varying pattern. The exceptions to the strong association with ASD were: learning difficulties had the strongest impact on *Language acquisition* and *Semantic-pragmatic skills* while SLI was associated with *Articulation*. The consistency of the associations for individual measures probably reflected their selection to predict ASD. It is interesting to note that the four individual measures mapped onto the four factors most specific to ASD.

**Table 5:** Linear regression analysis of traits with ASD and other co-morbid conditions adjusting for gender.

Trait	Max	ASD		SLI		Learning Difficulties		ADHD		ODD/CD		Anxiety problems		SEN	
	Effect	B	p	B	p	B	p	B	p	B	p	B	p	B	p
1: Verbal ability	ASD	−3.04	<0.001	−1.95	<0.001	−1.12	<0.001	−0.46	<0.001	−0.07	0.231	−0.24	<0.001	−1.30	<0.001
2: Language acquisition	LD	−0.16	0.134	−0.30	<0.001	−0.42	<0.001	0.18	0.012	0.30	<0.001	0.31	<0.001	−0.23	<0.001
3: Social understanding	ASD	−1.92	<0.001	−0.13	0.037	−0.36	<0.001	−1.20	<0.001	−1.28	<0.001	−0.45	<0.001	−0.43	<0.001
4: Semantic-pragmatic skills	LD	−0.92	<0.001	−0.01	0.851	−1.59	<0.001	−0.81	<0.001	−0.63	<0.001	−0.40	<0.001	−0.62	<0.001
5: Repetitive-stereotyped	ASD	−2.93	<0.001	−0.51	<0.001	−0.29	<0.001	−1.12	<0.001	−0.79	<0.001	−1.07	<0.001	−0.60	<0.001
6: Articulation	SLI	−0.41	<0.001	−1.46	<0.001	0.13	0.105	−0.44	<0.001	−0.24	<0.001	−0.19	0.002	−0.31	<0.001
7: Social inhibition	ASD	−1.16	<0.001	−0.34	<0.001	−0.04	0.645	0.38	<0.001	0.39	<0.001	−0.08	0.183	−0.17	<0.001
Factor mean score	ASD	−1.50	<0.001	−0.67	<0.001	−0.53	<0.001	−0.50	<0.001	−0.33	<0.001	−0.30	<0.001	−0.52	<0.001
CCC – coherence 9y	ASD	−7.36	<0.001	−3.44	<0.001	−2.77	<0.001	−2.68	<0.001	−1.40	<0.001	−1.38	<0.001	−2.71	<0.001
SCDC 91 m	ASD	−10.04	<0.001	−2.82	<0.001	−2.91	<0.001	−8.92	<0.001	−8.20	<0.001	−4.11	<0.001	−3.27	<0.001
Repetitive behaviour 69 m	ASD	−1.10	<0.001	−0.28	<0.001	−0.23	<0.001	−0.41	<0.001	−0.34	<0.001	−0.28	<0.001	−0.29	<0.001
EAS – Sociability 38 m	ASD	−3.41	<0.001	−1.00	<0.001	−0.24	0.326	0.41	0.076	0.49	0.009	−0.14	0.470	−0.59	<0.001
Prevalence			0.6%		3.0%		1.8%		2.1%		3.2%		3.1%		6.6%
N			13138		8282		7354		8222		8222		8253		10855
ASD cases			80		49		21		34		34		58		73
Prevalence			100.0%		59.2%		14.3%		35.3%		20.6%		22.4%		98.6%

See Methods S3 for definitions of the abbreviations associated with the individual measures and the co-morbid conditions.

All traits were standardised to have a variance of one. The data for traits included observed and imputed data.

**Figure 2:**
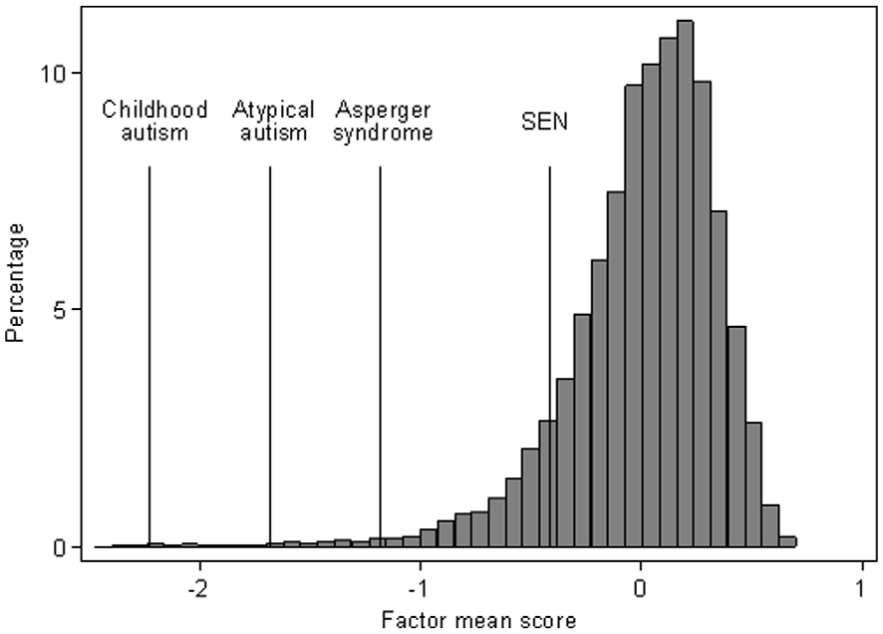
Distribution of the mean score for the 7 factors derived from the 93 traits. ASD children tended to have low scores on average with those classified as Childhood autism having worse scores than Asperger children. Other children with special educational needs also had worse scores than the general population but their deviation from this average was relatively minor.

As a further illustration of the specificity of these traits, the distribution of the factor mean score with the average locations of ASD diagnostic groups and other SEN children is shown in Figure 2. It can be seen that children classified with childhood autism had the worst scores with those with Asperger's syndrome having better scores although still somewhat worse than the population norm of zero. SEN children also had worse scores on average but the deviation from the norm was relatively minor.

### Genetic correlates

**Figure 3:**
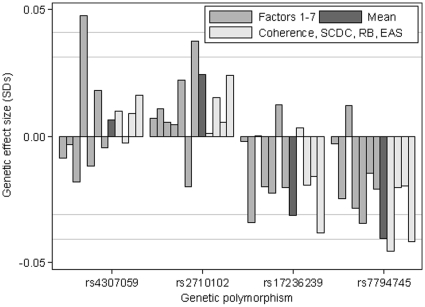
Genetic associations with the traits. The figure shows the associations for the four genetic markers with the traits (N = 7959–8436 for factors and Factor mean score; 7324–7760 for individual measures). Effect sizes are reported as a proportion of a SD for each outcome for each copy of the minor allele. Approximate 5% and 1% grid lines are shown.

In order to investigate the extent to which factors may reflect the operation of different aetiological processes, we examined the association between the factors and four SNPS with common genetic variants that have been previously associated with ASD. Major alleles of the cadherin (rs4307059; *CDH9/CDH10*) and contactin (rs2710102; *CNTNAP2*) SNPs were associated with worst scores on factor 4 (p = 0.005) and factor 7 (p = 0.017) respectively (see Figure 3). In contrast, minor alleles of the other contactin SNPs (rs17326239 and rs7794745; *CNTNAP2*) were associated with worse scores for Factor 2 (rs17236239 only, p = 0.028), the Factor mean score (p<0.043), *CCC – coherence 9y* (rs7794745 only, p = 0.009) and *EAS – sociability 38 m* (p<0.023). These results provided some evidence of heterogeneity with markers from different genes being associated with different traits. In addition there was also support for pleiotropic effects whereby different markers from the same gene were associated with a range of traits.

### Age of diagnosis

Age of ASD diagnosis (N = 66) was positively associated with trait scores in linear regression analyses for Factors 1, 5 and 7 (p<0.003) and for the four individual measures (p<0.020), not associated for Factors 2 to 4 and negatively associated with Factor 6 (p = 0.016). The positive associations may reflect that later diagnoses are linked to milder forms of ASD rather than early diagnoses increasing awareness and hence reporting of the traits. The negative association for Factor 6 (*Articulation*) may indicate that deficits on this trait tend to precipitate a SLI diagnosis and it is only after persistent problems that the diagnosis is changed to ASD.

## Discussion

This study identified seven orthogonal factors that reflected a number of putative component ASD traits. These included verbal ability, language acquisition, semantic-pragmatic skills, social understanding, repetitive-stereotyped behaviour, articulation and social inhibition. All were related to ASD outcome.

We identified more factors than in previous reports for a number of reasons. First, the large sample size of this study compared to previous investigations provided extra power to detect more minor factors. Second, this was a population based cohort in which measures were collected at different points in development. This helped to identify less major factors partly because the sample encompassed the full range of responses compared to clinical samples but also because the use of repeat measures helped to increase the proportion of variability in the data associated with such factors. Finally, we included a wide range of measures in this study. In contrast, some previous studies only analysed composite scores rather than the individual measures, for instance, the 12 subscales of the Autism Diagnostic Interview – Revised diagnostic instrument [13], [14]. This may have limited their scope to detect multi-factorial solutions. But it is important to note that some differences are attributable to the method chosen to identify the number of factors. In this study, we found a wide range of possible solutions based upon different criteria but chose the seven factors based upon parsimony and interpretability. Other studies may have also identified a larger number of factors but chosen to interpret this as a fewer number based upon a single criterion such as variance explained before rotation [11].

The factors we identified showed some similarities to the factors reported in two previous studies [15], [43]. For instance, the identification of language milestones and the role of imaginative play has been not been frequently reported but is consistent with Factor 2 in this study. However both of these studies differentiated between different aspects of repetitive behaviour and restricted interests not found in this study. This may reflect the fact that there were comparatively few measures of this latter type (e.g. insistence on sameness) included in this study. The most consistent findings across studies concerned the identification of factors pertaining to social-communication and repetitive interests and behaviours [9], [12]–[15], [43], [44]. This study also identified factors relating to these major domains of function, although our findings indicated that within the main domains, there was evidence for further fractionation of the phenotype, with 4 factors related to communication, two with social and one with repetitive domains. Despite these overall consistencies, differences in the detailed factor structures from previous studies were observed [9]. These differences might be attributed in part to their cross-sectional nature and the possibility that their data reflected transient states. Our longitudinal study was in a stronger methodological position to identify the more enduring traits which might be expected to produce a more stable and reproducible factor structure.

All seven factors were independently associated with ASD diagnosis and the combined factor score showed a high sensitivity to diagnostic status, reflecting the cumulative contribution of the individual factors to diagnosis. The individual factor scores did not predict ASD status as well as some of the individual measures. This may reflect the fact that the individual measures that best predicted an ASD diagnosis (e.g. the CCC scores) were often specifically developed to measure ASD traits. Moreover, some of these individual measures were collected after the child had been diagnosed with ASD, so they may have been subjected to more reporting bias. The approach we have adopted here of relating factor scores and individual measures to ASD status has the advantage of helping to identify those measures that may be most informative for future research from amongst the wide number of putative traits available. This approach can help to circumvent the problems of multiple testing that arise when investigating aetiological determinants of the richly characterized and complex phenotypes observed in large data sets such as ALSPAC.

Previous research has suggested that different components of the ASD phenotype may have different aetiological origins [8]. While this study has shown that a number of traits, whether individual measures or derived measures from factor analysis, have independent contributions to the diagnosis of ASD which adds support to this hypothesis, in practice, this may not be sufficient. Some have argued that such traits may have more association with obtaining a diagnosis than the underlying biological processes [45]. As a further exploration of this issue, the associations of the identified factors and individual measures with four genetic correlates within the cadherin and contactin genes were examined. Different genetic variants were associated with different factors – in particular Factor 2 (*Language acquisition*), Factor 4 (*Semantic-pragmatic skills*), Factor 7 (*Social inhibition*) and the Factor mean score. The results partially replicate previous reports from studies of individuals with ASD, where associations were reported for age at first word and expressive language, but also extend their findings [17], [18]. While pleiotropic effects may contribute to some of the heterogeneity in the ASD phenotype [46], as observed in this study for the contactin variants, the contrast in results with the cadherin variant favoured a broader phenotype with differentiable components and more complex aetiological origins.

A recent study related the same cadherin SNP with 29 measures encompassing language, communication, social interaction and behavioural traits [47]. Consistent associations were observed with only one measure showing an effect opposite to the expected direction. In contrast, we found one out of 4 individual measures and 5 out of 7 factors with this unexpected direction to the best estimate of the effect size. While that study found a significant joint association even amongst those traits with weaker associations, our results, ignoring Factor 4 (*semantic-pragmatic skills*), are more consistent with a null association overall and may re-enforce the conclusion that our identified traits, especially the factors, encompass greater heterogeneity. The strong association for Factor 4 is consistent with that study's report of an association with *CCC – stereotyped conversation 9y*.

It was notable that the analyses of measures taken at different points in development supported the notion that the phenotypic architecture of the broader autism phenotype unfolds and becomes more differentiated with development. The implication is that aetiological studies need to take these developmental changes into consideration and recognize that genetic and environmental influences may operate developmentally and may differ in importance at different ontological stages.

This study has also shed light on some statistical issues. Some debate has occurred on whether oblique or orthogonal rotation should be used in factor analyses [48]. While it is true that oblique rotations can produce orthogonal factors if appropriate to the data, it is clear from our study that relatively high correlations between oblique factors may result from relatively marginal changes to the factor structure. Our study also showed that an overall orthogonal association does not necessarily imply orthogonality at the worst extremes of the factor scores where pathology may be most evident. Overall, these findings may detract from the theoretical advantages of oblique rotation methods and favour orthogonal methods especially in population-based samples. It has also been suggested that the variance explained by the retained factors should usually be less than 100% [49]. While some consider that the presence of negative eigenvalues implies that the positive eigenvalues are overestimated and even to retain factors explaining 100% of the variance would be an over-factorisation, others see the negative eigenvalues as a facet of underestimating the communalities [50]. It is difficult to generalise from our study, but the presence of a single factor explaining 108% of the variance found in one analysis suggests that underestimation of communalities should not be discounted.

This study has some potential limitations. The individual measures accessed from the ALSPAC database were in general not specifically designed to assess ASD. While this strategy of including questions for a range of health and developmental outcomes may have omitted some traits more specific to ASD, our results suggest a significant portion of the variability associated with ASD has been explained. Self-completed questionnaires were the major source of data with 88 of the 93 individual measures being obtained in this way. This contrasts with diagnostic tests, such as the Autism Diagnostic Observation Schedule – Generic or the Autism Diagnostic Interview – Revised, which require trained personnel. Despite this potential limitation, maternal reporting has been shown to have high sensitivity for detecting global developmental deficits [51]. Finally, many of the standard measures were abbreviated for pragmatic reasons. While this raises concerns over their comparability with the full form, such short forms have been shown to have acceptable reliability eg [52].

In summary, this study has identified seven factors reflecting aspects of communication encompassing early language development and later verbal ability, semantic-pragmatic skills, and articulation patterns; difficulties in social understanding and inhibition; and repetitive-stereotyped behaviour. Individual measures were also identified some of which retained predictive power even in the presence of these factors.

We conclude that the evidence from these analyses lend support to the notion that the main traits associated with ASD both theoretically and empirically (social, communication and repetitive behaviours) need to be considered as potentially distinct components of the ASD phenotype, with their own as well as shared genetic and environmental determinants. Equally it needs to be borne in mind, that some of the traits identified here may not be core components of the ASD phenotype but, nevertheless, shape elements of the manifestations of the syndrome.

## Supporting Information

Methods S1 (0.04 MB DOC)

Methods S2 (0.04 MB DOC)

Methods S3 (0.02 MB DOC)

Results S1 (0.03 MB DOC)

Table S1 Standard measures used in this study.
(0.06 MB DOC)

Table S2 Means and SDs of observed and imputed data.
(0.15 MB DOC)

Table S3 Maximum and estimated communalities for individual measures using different data sets.
(0.19 MB DOC)

Table S4 Comparisons of factor loadings generated for different data sets compared to the results for the standard imputed data set.
(0.05 MB DOC)

Table S5 Factors analyses of individual measures classified by age.
(0.17 MB DOC)

Table S6 Prevalence of the worst decile calculated for each trait by ASD status and type of data.
(0.18 MB DOC)

Table S7 Subset regression results of 93 individual measures on ASD status adjusting for gender.
(0.05 MB DOC)

Figure S1 Scree plots from factor analyses of individual measures associated with each factor. While a combined analysis of all 93 measures has identified the major factors, there was evidence that more minor factors existed in a hierarchical structure (see Table 1, Figure 1). These minor factors may be more apparent in separate analyses of measures associated with a single factor rather than in combined analyses. In part A, the factor structure of measures associated with factors 1 to 3 was not further differentiated. But analysis of measures associated with factors 4 to 7 in part B, showed the possibility of more minor factors. The factor structure became differentiated with duplicate measures clustering on the same factor. The definition of ‘duplicate’ varied between factors. Hence for the analysis of Factor 4, the split was by questionnaire/clinic measures. For other factors, different questions formed different factors with repeat measures clustering on the same factor. In particular for Factor 5, DAWBA measures clustered on the same factor. These four major factors might be separated into 10 minor factors.
(0.03 MB TIF)

Figure S2 Scree plots from factor analyses of individual measures relating to different age ranges. Analysis of 8 traits at 6–15 m and 22 traits at 18–38 m (part A) and, 31 traits at 42–77 m and 32 traits at 81 m–9y (part B) suggested 1, 5, 6 and 7 factors respectively.
(0.03 MB TIF)
