# Supplementary material for: Traits Contributing to the Autistic Spectrum
Source: PLoS One. 2010 Sep 8;5(9):e12633. doi: 10.1371/journal.pone.0012633 (PMC2935882; doi:10.1371/journal.pone.0012633)
Supplement: Methods S1 — (0.04 MB DOC) [file pone.0012633.s001.doc]

**Methods S1**

*Description of the individual measures*

Of the 93 traits, 46 related to standard measures listed in Table S1. Many of these tests were abbreviated, adapted or subscales modified in order to make the collection of data practical in such a large cohort. For instance, a number of revisions were made to the MacArthur CDI [1]. In Part I of *Words and Gestures*, only 10 semantic categories out of the 19 were utilised with a further selection of the 234 words listed in these categories. Vocabulary understanding and production were combined into a single score to be consistent with other measures which incorporated both receptive and expressive facets of language. In addition, a number of the subscales related to imitation games, role-playing and imaginative games from Part II were combined into a single score due to the overlap in characteristics. At 38 months for *Words and Sentences*, 12 questions concerning the child’s use of inflections and closed class words were asked. While relating to the level of sentence complexity and knowledge of syntax, these questions also considered examples of over-regularisation (eg *two foots*). Hence these two subscales were combined. Further details about the specific questions can be found on the ALSPAC website [2].

For other measures of shorter length, the relevant subscales were implemented in their full form. These included the Rutter scale, SDQ, DANVA, SCDC, CCC [3-7]. (Definitions of these abbreviations and others used in this paper are given in Methods S3.)

Of the 93 individual measures, 47 related to questions specifically designed for ALSPAC questionnaires. In most cases, these were asked four times between 38 and 81 months with possible responses being *never*, *sometimes*, *often* and/or *always*.

1. Intelligibility

A score was derived from three questions relating to whether the parents, relatives or friends/visitors can understand the child’s speech.

1. Stumbles on words

The question in more detail was *Does your child stumble or get stuck on words, or repeat them many times? (e.g. I I I I want a sweet)*.

1. Echoes what said

The specific question was *Does your child* *echo what has just been said to her (e.g. you say; 'we are going out now' she says: 'going out now')*

1. Stays mainly silent
2. Avoids eye contact
3. Prefers gestures

This question related to whether child preferred to use pointing or pulling gestures to get something rather than asking for it with words. Responses were *never*, *yes but not now* or *yes still does*.

1. Pronouncing certain sounds (57m to 81m)

This question related to certain sounds such as *th*, *sss* or *t* .

1. Repetitive behaviour

A score was derived from three questions relating to whether the child repeatedly rocks head or body, has tics or twitches or other unusual behaviour. These questions were asked six times between 18 months and 77 months. At 42 months, a fourth question from the Rutter scale on tics, mannerisms or twitches was also included.

1. Nonverbal communication (81m only)

A score was derived from questions concerning the child’s ability to recognize other people’s feelings from facial expressions or the tone of their voice.

1. Communication

This score reflected a number of questions concerning listening attentively, understanding instructions, talking clearly and asking sensible questions.

1. Musical (57m to 81m)

This score assessed the abilities to sing songs, hum a tune or clap in time to music.

1. Empathy (57m to 81m)

This score assessed the child’s willingness to share toys, take turns in games, please the parents and to feel sympathy when others are hurt.

Items (i) to (vi) were adapted and designed for use in ALSPAC questionnaires with advice from Sue Roulstone, Sue Loader and Professor Sir Michael Rutter. Items (ii) to (vii) above and *DAWBA – tics or twitches* related to single questions while the other 69 individual measures related to derived scores from multiple questions.

Nearly all of the data for individual measures were obtained from postal questionnaires completed by the main carer. Only the five measures at 8y, *WOLD – comprehension*, *WOLD – oral expression*, *Nonword repetition*, *WISC – verbal IQ* and *DANVA – faces*, were assessed in a special clinic where assessments were made by trained personnel or involved computerised data entry.

All scores were coded so that high scores reflected a favourable performance. In practice this led to responses from the DAWBA, SCDC and items (i) to (viii) above being reversed. In general the interpretations of these variables are unambiguous eg high scores on *Social Fears* imply a lack of social fears. But the use of gestures may require some further clarification. At 15 months, gestures are a normal part of development and high scores should be interpreted as frequent use of gestures to convey meaning. At later ages, gestures play a more minor role compared to expressive language. Hence, high scores on *Prefers Gestures* would indicate infrequent use of gestures with these children preferring to use words to convey meaning.

**References**
