# Supplementary material for: Traits Contributing to the Autistic Spectrum
Source: PLoS One. 2010 Sep 8;5(9):e12633. doi: 10.1371/journal.pone.0012633 (PMC2935882; doi:10.1371/journal.pone.0012633)
Supplement: Methods S2 — (0.04 MB DOC) [file pone.0012633.s002.doc]

**Methods S2**

*Parallel Analysis*

Parallel analysis estimates the null distribution of each ordered eigenvalue from simulated data [1]. In this study, we chose an alternative approach whereby the observed data was randomly permutated. This strategy has the advantage of preserving the actual distributions of each variable rather than imposing an assumed distribution as in simulated data. The decision on the number of factors should be based upon those observed eigenvalues which exceed the ‘parallel’ eigenvalue as derived from the random data, or in other words, what might be expected by chance. This method has been shown to be one of the best methods for selecting the number of factors being superior to other methods such as the scree plot and Kaiser’s K1 criterion [2]. A modification to this technique uses the 95^th^ centile (rather the median) of the null distributions to compensate for the tendency to select too many factors [3]. However, while this technique works well with principal component analysis (PCA), its application to principal factor analysis (PFA) is less clear [4]. The fundamental difference is that the sum of the eigenvalues is the same for the observed and random data in PCA while in PFA they will differ substantially. A correction has been proposed whereby the critical values should be inflated by the average difference in observed/random eigenvalue totals [4]. In this study, the appropriate correction was 0.452.

*Goodness-of-fit statistics*

The model χ^2^ statistic is one of the most widely quoted statistic but its limitations, in particular its association with sample size, make it difficult to assess whether significant ‘lack of fit’ indicates a poor fit in a practical sense or just minor differences detectable with a large sample. Other statistics, such as RMSEA and CFI, attempt to adjust the χ^2^ values to achieve indications of a good, reasonable or poor fit [5]. While this helps the interpretation, it is also useful to examine the residual correlations between the observed and predicted correlation matrices. This may reveal pockets of poor fit despite an overall reasonable fit [6,7].

Another common statistic is the cumulative variance explained by successive numbers of factors [8]. Usually in principal factor analysis, the explanation is expressed as a percentage of the sum of the communalities (that is, the trace of the modified correlation matrix) rather than the sum of the total variance (equivalent to the number of variables for analyses of the correlation matrix). This percentage can exceed 100% due to the presence of negative eigenvalues although in practice it is discouraged to consider factors in this range [9].

**References**

1. Horn JL (1965) A rationale and test for the number of factors in factor analysis. Psychometrika 30: 179-185.

2. Hayton JC, Allen DG, Scarpello V (2004) Factor retention decisions in exploratory factor analysis: A tutorial on parallel analysis. Organ Res Methods 7: 191-205.

3. Glorfeld LW (1995) An improvement on Horns parallel analysis methodology for selecting the correct number of factors to retain. Educ Psychol Meas 55: 377-393.

4. Buja A, Eyuboglu N (1992) Remarks on parallel analysis. Multivar Behav Res 27: 509-540.

5. Hu L, Bentler PM (1999) Cutoff criteria for fit indexes in covariance structure analysis: Conventional criteria versus new alternatives. Struct Equ Modeling 6: 1 - 55.

6. MacCallum RC (2003) Working with imperfect models. Multivar Behav Res 38: 113-139.

7. Kline RB (2005) Principles and practice of structural equation modeling. New York: Guilford Press.

8. Jackson DA (1993) Stopping rules in principal components analysis: a comparison of heuristic and statistical approaches. Ecology 74: 2204-2214.

9. Harman HH (1976) Modern factor analysis. Chicago, IL: University of Chicago Press.
