# Supplementary material for: Traits Contributing to the Autistic Spectrum
Source: PLoS One. 2010 Sep 8;5(9):e12633. doi: 10.1371/journal.pone.0012633 (PMC2935882; doi:10.1371/journal.pone.0012633)
Supplement: Methods S3 — (0.02 MB DOC) [file pone.0012633.s003.doc]

# Methods S3

# *Abbreviations*

# ALSPAC Avon Longitudinal Study of Parents and Children

ADHD Attention deficit hyperactivity disorder

ASD Autistic Spectrum Disorder

# CCC Children’s Communication Checklist

# CDI Communicative Development Inventory

CFI Comparative Fit index

DANVA Diagnostic Analysis of Nonverbal Accuracy scale

# DAWBA Development and Well-Being Assessment

DDST Denver Developmental Screening Test

# EAS Emotionality, Activity and Sociability Temperament scale

ODD/CD Oppositional defiant/conduct disorders

PCR Polymerase chain reaction

PLASC Pupil Level Annual School Census

RMSEA Root mean square error of approximation

SCDC Social and Communication Disorders Checklist

SDQ Strength and Difficulties Questionnaire

SEN Special Educational Needs

SLI Specific language impairment

SNP Single Nucleotide Polymorphism

SRMR Standardised root mean square residual

# WISC Wechsler Intelligence Scale for Children

# WOLD Wechsler Objective Language Dimensions
