# Supplementary material for: Traits Contributing to the Autistic Spectrum
Source: PLoS One. 2010 Sep 8;5(9):e12633. doi: 10.1371/journal.pone.0012633 (PMC2935882; doi:10.1371/journal.pone.0012633)
Supplement: Results S1 — (0.03 MB DOC) [file pone.0012633.s004.doc]

**Results S1**

*Supplementary factor analyses*

To investigate how sensitive the results from the imputed data set might be to data characteristics, such as imputation, particular subgroups of the whole population and repeat measures, analyses were repeated for different data sets (see Table S3).

The factor structure appeared resilient to the imputation process. Repeating the factor analysis using pair-wise correlations (N=5201 to 11316) or using complete data only (N=2481) showed similar results. Also, introducing extra variables, such as aspects of family adversity (eg parental conflict and alcohol abuse), into the imputation process had little impact on the results despite their associations with the presence/absence of data.

Factor analyses were also repeated for particular subgroups to investigate the uniformity of the factor structure across the full population range. Full-scale IQ was used as a measure of the variability within this sample with children in the bottom and top quartiles of IQ being analysed separately. Discrepancies between these results and the full sample results were noted particularly for Factors 4 and 6. While some of these differences might be attributed to the reduced sample size in these secondary analyses, it appeared that the 8y measures and *Prefers gestures* (at 4 ages) were no longer loading on their respective factors. However, in the former case, stratification by IQ, another 8y measure, would have reduced the systematic variability in these contemporary measures which would have contributed to the low loadings. This was reflected in the lower communality estimates (see Table S3). For *Prefers gestures*, the discrepancies reflected loadings just above or below the 0.3 criterion rather than any major difference in loadings. In addition, further discrepancies were noted on Factors 1 and 5 for those in the high IQ group only. For this subgroup, the associated individual measures loaded more strongly on CDI measures at 24m and less strongly on CCC measures and measures of *Repetitive behaviour* (18–77m). This may reflect the earlier development of communication skills in this subgroup and the highly skewed nature of the CCC and *Repetitive behaviour* measures producing less discrimination at the favourable extremes of the scales.

The impact of repeat measures on the factor structure was also investigated. It was noted that these measures tended to cluster together which may have reflected the enduring nature of particular traits. On the other hand, it is possible that the correlations between repeat assessments by the same rater were inflated by between rater variability with some tending to score consistently more severely while others more leniently. In practice, both explanations are likely to contribute to the correlation. However, since no single factor was strongly associated with only one set of repeat measures, it was expected that the exclusion of repeat measures may not have produced a similar factor structure. However, in practice, this data set had the most extreme deviations from the standard data set. This may have been a facet of over-factorisation reflected in estimated communalities exceeding the ‘maximum’ (see Table S3) [1]. It may also have indicated that with fewer measures, the more minor facets of the factor structure as shown in Figure S1 became more prominent and as a consequence, seven factors was not an adequate summary of the data. Finally, as shown in Table S3, the communalities for some individual measures were substantially reduced – in particular for measures associated with Factors 6 and 7. This would have limited their ability to load highly on any factor.

**Reference**

1. Harman HH (1976) Modern factor analysis. Chicago, IL: University of Chicago Press.
