# Supplementary material for: Traits Contributing to the Autistic Spectrum
Source: PLoS One. 2010 Sep 8;5(9):e12633. doi: 10.1371/journal.pone.0012633 (PMC2935882; doi:10.1371/journal.pone.0012633)
Supplement: Table S1 — Standard measures used in this study. (0.06 MB DOC) [file pone.0012633.s005.doc]

Table S1: Standard measures used in this study

| Measure | Subscale | Score Identifier | Age range ^a^ |
| --- | --- | --- | --- |
| Denver Developmental | Communication | Communication | 6m, 15m |
| Screening test |  |  |  |
| MacArthur Infant | Vocabulary Understanding | Vocabulary | 15m |
| Communicative Development | Vocabulary Production |  |  |
| Inventories (CDI) | First signs of understanding | Response to language | 15m |
| (Words and Gestures) | Understands phrases | Understands | 15m |
|  | Initiating words/labelling objects | Imitates words | 15m |
|  | First communicative gestures | Gestures | 15m |
|  | Actions with objects | Objects | 15m |
|  | Games and routines | Pretend Play | 6m – 42m |
|  | Pretending to be a parent |  |  |
|  | Initiating other adult actions |  |  |
|  | Imaginative play |  |  |
| MacArthur Toddler CDI | Vocabulary Production | Vocabulary ^c^ | 24m, 38m |
| (Words and sentences) ^b^ | Use of suffixes | Grammar (regular) | 24m, 38m |
|  | Irregular nouns and verbs | Grammar (irregular) | 24m, 38m |
|  | Overregularisations | Complexity | 38m |
|  | Sentence complexity |  |  |
|  | Length of utterance | Combines words | 24m – 81m |
| EAS Temperament | Sociability | Sociability | 38m ­– 69m |
| Rutter Parent Scale | Prosocial | Prosocial | 42m |
| Strengths and Difficulties | Prosocial | Prosocial | 47m – 9y |
| Development and Well- | Compulsions | Number Compulsions | 91m |
| Being Assessment |  | Compulsions score | 91m |
| (DAWBA) ^d^ | Social fears | Social fears | 91m |
|  | Tics | Tics or twitches | 91m |
| Social Communication |  | SCDC | 91m |
| Disorders Checklist ^d^ |  |  |  |
| WISC | Verbal | Verbal IQ | 8y |
| Wechsler Objective | Comprehension | Comprehension | 8y |
| Language Dimensions | Oral Expression | Oral Expression |  |
| Nonword Repetition |  | Nonword repetition | 8y |
| Diagnostic Analysis of | Faces | Faces | 8y |
| Nonverbal Accuracy ^e^ |  |  |  |
| Children’s Communication | Intelligibility & fluency | Speech | 9y |
| Checklist (CCC) ^d, f^ | Syntax | Syntax | 9y |
|  | Inappropriate initiation | Inappropriate initiation | 9y |
|  | Coherence | Coherence | 9y |
|  | Stereotyped conversation | Stereotyped conversation | 9y |
|  | Use of conversational context | Use of context | 9y |
|  | Conversational rapport | Rapport | 9y |

^a^ Age range for multiple assessments. Average age of children attending the 8y clinic was 8y 8m. The 9y questionnaire had a target age of 9y 7m.

^b^ *Decontextualised language* was not assessed.

^c^ Vocabulary score includes credit for understanding only.

^d^ Scores have been reversed so that high scores indicate a favourable response.

^e^ Data for the *Voices* subtest is not currently available.

^f^ *Social relationships* and *Interests* were not assessed.
