# Supplementary material for: Traits Contributing to the Autistic Spectrum
Source: PLoS One. 2010 Sep 8;5(9):e12633. doi: 10.1371/journal.pone.0012633 (PMC2935882; doi:10.1371/journal.pone.0012633)
Supplement: Table S2 — Means and SDs of observed and imputed data. (0.15 MB DOC) [file pone.0012633.s006.doc]

Table S2: Means and SDs of observed and imputed data (N=13138)

| Age | Trait | Max ^a^ | Observed | | | Imputed | | | p ^b^ |
| --- | --- | --- | --- | --- | --- | --- | --- | --- | --- |
|  |  |  | N | Mean | SD | N | Mean | SD |  |
| 6m | DDST – Communication | 16 | 11346 | 11.12 | 1.96 | 1792 | 11.23 | 0.61 | <0.001 |
|  | Pretend play | 4 | 11324 | 1.77 | 1.41 | 1814 | 1.83 | 0.39 | <0.001 |
| 15m | CDI – understand score | 12 | 11031 | 9.13 | 2.47 | 2107 | 9.19 | 1.12 | 0.073 |
|  | CDI – Vocabulary | 268 | 11020 | 87.48 | 45.76 | 2118 | 89.00 | 25.93 | 0.032 |
|  | CDI – response to language | 4 | 11030 | 3.77 | 0.49 | 2108 | 3.76 | 0.11 | 0.237 |
|  | CDI – imitates words | 4 | 11013 | 2.10 | 1.17 | 2125 | 2.12 | 0.53 | 0.081 |
|  | CDI – gestures | 20 | 10959 | 13.94 | 3.40 | 2179 | 14.05 | 1.58 | 0.018 |
|  | CDI – objects | 32 | 10976 | 17.32 | 5.67 | 2162 | 17.43 | 2.82 | 0.179 |
| 18m | DDST – communication | 28 | 11106 | 15.93 | 4.79 | 2032 | 16.20 | 2.92 | 0.001 |
|  | Pretend play | 6 | 11082 | 5.52 | 0.83 | 2056 | 5.52 | 0.30 | 0.660 |
| 24m | CDI – Vocabulary | 246 | 10300 | 152.08 | 56.40 | 2838 | 152.69 | 37.84 | 0.502 |
|  | CDI – grammar (regular) | 8 | 10300 | 3.44 | 2.56 | 2838 | 3.49 | 1.48 | 0.211 |
|  | CDI – grammar (irregular) | 50 | 10300 | 18.85 | 13.77 | 2838 | 19.49 | 8.05 | 0.002 |
|  | CDI – combines words | 2 | 10049 | 1.36 | 0.77 | 3089 | 1.31 | 0.42 | <0.001 |
| 30m | Pretend play | 14 | 10267 | 6.50 | 2.33 | 2871 | 6.57 | 1.18 | 0.044 |
| 38m | CDI – Vocabulary | 246 | 10139 | 228.14 | 32.70 | 2999 | 225.76 | 18.10 | <0.001 |
|  | CDI – grammar (regular) | 8 | 9905 | 6.73 | 1.81 | 3233 | 6.42 | 1.24 | <0.001 |
|  | CDI – grammar (irregular) | 50 | 10139 | 39.94 | 12.11 | 2999 | 39.56 | 5.82 | 0.018 |
|  | CDI – complexity | 24 | 10139 | 19.98 | 5.30 | 2999 | 19.55 | 2.65 | <0.001 |
|  | CDI – combines words | 4 | 9950 | 3.90 | 0.40 | 3188 | 3.83 | 0.28 | <0.001 |
|  | Communication | 10 | 10023 | 8.52 | 1.30 | 3115 | 8.33 | 0.83 | <0.001 |
|  | Intelligibility | 6 | 10018 | 5.61 | 0.88 | 3120 | 5.49 | 0.53 | <0.001 |
| 42m | Pretend play | 16 | 10046 | 9.44 | 2.43 | 3092 | 9.48 | 1.31 | 0.320 |
| 57m | Communication | 12 | 9452 | 11.75 | 0.87 | 3686 | 11.57 | 0.88 | <0.001 |
|  | Musical | 8 | 9443 | 7.39 | 1.16 | 3695 | 7.30 | 0.68 | <0.001 |
|  | Intelligibility | 9 | 9463 | 7.84 | 2.38 | 3675 | 7.74 | 0.73 | <0.001 |
|  | Combines words | 5 | 9458 | 4.91 | 0.39 | 3680 | 4.85 | 0.28 | <0.001 |
| 69m | Communication | 12 | 8647 | 11.83 | 0.71 | 4491 | 11.70 | 0.65 | <0.001 |
|  | Musical | 8 | 8639 | 7.68 | 0.86 | 4499 | 7.61 | 0.50 | <0.001 |
|  | Intelligibility | 9 | 8654 | 7.91 | 2.58 | 4484 | 7.83 | 0.64 | 0.004 |
|  | Combines words | 5 | 8641 | 4.95 | 0.31 | 4497 | 4.91 | 0.21 | <0.001 |
| 81m | Communication | 12 | 8440 | 11.86 | 0.65 | 4698 | 11.72 | 0.64 | <0.001 |
|  | Musical | 8 | 8449 | 7.74 | 0.76 | 4689 | 7.67 | 0.44 | <0.001 |
|  | Intelligibility | 9 | 8450 | 8.07 | 2.44 | 4688 | 7.96 | 0.64 | <0.001 |
|  | Combines words | 5 | 8429 | 4.97 | 0.26 | 4709 | 4.93 | 0.19 | <0.001 |
| 9y | CCC – intelligibility & fluency | 22 | 8146 | 19.25 | 1.99 | 4992 | 18.99 | 1.44 | <0.001 |
|  | CCC – syntax score | 8 | 8118 | 7.82 | 0.62 | 5020 | 7.71 | 0.54 | <0.001 |
|  | CCC – coherence | 16 | 8120 | 14.81 | 2.08 | 5018 | 14.55 | 1.47 | <0.001 |
| 42m | Rutter Prosocial | 22 | 10032 | 15.33 | 3.62 | 3106 | 15.28 | 1.88 | 0.345 |
| 47m | SDQ Prosocial | 10 | 9534 | 7.04 | 1.97 | 3604 | 7.02 | 1.06 | 0.532 |
| 57m | Empathy | 10 | 9457 | 9.13 | 1.20 | 3681 | 9.06 | 0.62 | <0.001 |
| 69m | Empathy | 10 | 8640 | 9.41 | 1.01 | 4498 | 9.33 | 0.55 | <0.001 |
| 81m | Empathy | 10 | 8456 | 9.40 | 1.00 | 4682 | 9.34 | 0.54 | <0.001 |
|  | SDQ Prosocial | 10 | 8442 | 8.17 | 1.76 | 4696 | 8.15 | 1.02 | 0.452 |
| 91m | SCDC ^c^ | 24 | 8131 | 21.16 | 3.73 | 5007 | 20.85 | 2.02 | <0.001 |
| 97m | SDQ Prosocial | 10 | 7796 | 8.02 | 1.90 | 5342 | 7.99 | 1.11 | 0.255 |
| 9y | SDQ Prosocial | 10 | 8082 | 8.32 | 1.66 | 5056 | 8.33 | 0.90 | 0.608 |
|  | CCC – conversational rapport | 16 | 7983 | 14.45 | 2.02 | 5155 | 14.29 | 1.16 | <0.001 |
| 38m | Echoes what said ^c^ | 2 | 9656 | 1.12 | 0.73 | 3482 | 1.10 | 0.26 | 0.001 |
| 57m | Echoes what said ^c^ | 2 | 8994 | 1.65 | 0.52 | 4144 | 1.61 | 0.22 | <0.001 |
| 69m | Echoes what said ^c^ | 2 | 8126 | 1.75 | 0.46 | 5012 | 1.70 | 0.21 | <0.001 |
| 81m | Echoes what said ^c^ | 2 | 8098 | 1.82 | 0.41 | 5040 | 1.77 | 0.17 | <0.001 |
|  | Nonverbal communication | 4 | 8211 | 3.41 | 1.03 | 4927 | 3.32 | 0.35 | <0.001 |
| 8y | WOLD – comprehension | 15 | 7383 | 7.46 | 1.96 | 5755 | 7.26 | 0.68 | <0.001 |
|  | WOLD – oral expression | 10 | 7350 | 7.44 | 1.83 | 5788 | 7.13 | 0.96 | <0.001 |
|  | Nonword repetition | 12 | 7367 | 7.23 | 2.51 | 5771 | 6.90 | 1.31 | <0.001 |
|  | WISC – verbal IQ | 155 | 7385 | 106.96 | 16.80 | 5753 | 103.89 | 9.55 | <0.001 |
|  | DANVA – faces | 24 | 6820 | 19.40 | 2.79 | 6318 | 19.25 | 0.94 | <0.001 |
| 9y | CCC – inappropriate initiation | 12 | 8115 | 8.72 | 2.43 | 5023 | 8.49 | 0.97 | <0.001 |
|  | CCC – stereotyped conversation | 16 | 8084 | 12.82 | 2.52 | 5054 | 12.55 | 1.09 | <0.001 |
|  | CCC – conversational context | 16 | 7989 | 13.75 | 2.16 | 5149 | 13.40 | 1.31 | <0.001 |
| 18m | Repetitive behaviour ^c^ | 6 | 11007 | 5.79 | 0.61 | 2131 | 5.75 | 0.21 | <0.001 |
| 30m | Repetitive behaviour ^c^ | 6 | 10221 | 5.90 | 0.46 | 2917 | 5.87 | 0.16 | <0.001 |
| 42m | Repetitive behaviour ^c^ | 8 | 9925 | 7.89 | 0.50 | 3213 | 7.86 | 0.21 | <0.001 |
| 57m | Repetitive behaviour ^c^ | 6 | 9388 | 5.91 | 0.40 | 3750 | 5.88 | 0.18 | <0.001 |
| 69m | Repetitive behaviour ^c^ | 6 | 8586 | 5.89 | 0.47 | 4552 | 5.85 | 0.22 | <0.001 |
| 77m | Repetitive behaviour ^c^ | 6 | 8426 | 5.90 | 0.46 | 4712 | 5.87 | 0.20 | <0.001 |
| 91m | DAWBA – Number Compulsions ^c^ | 8 | 8121 | 7.86 | 0.51 | 5017 | 7.81 | 0.19 | <0.001 |
|  | DAWBA – Compulsions score ^c^ | 16 | 8120 | 15.82 | 0.70 | 5018 | 15.76 | 0.28 | <0.001 |
|  | DAWBA – Tics or twitches ^c^ | 1 | 8054 | 0.98 | 0.14 | 5084 | 0.97 | 0.03 | 0.004 |
| 38m | Stumbles on words ^c^ | 2 | 9924 | 1.43 | 0.60 | 3214 | 1.42 | 0.16 | 0.808 |
|  | Prefers gestures ^c^ | 2 | 9973 | 1.19 | 0.58 | 3165 | 1.16 | 0.24 | <0.001 |
| 57m | Stumbles on words ^c^ | 3 | 9427 | 2.61 | 0.56 | 3711 | 2.58 | 0.22 | <0.001 |
|  | Prefers gestures ^c^ | 2 | 9454 | 1.51 | 0.57 | 3684 | 1.48 | 0.24 | <0.001 |
|  | Pronouncing certain sounds ^c^ | 1 | 9388 | 0.72 | 0.45 | 3750 | 0.72 | 0.16 | 0.852 |
| 69m | Stumbles on words ^c^ | 3 | 8633 | 2.74 | 0.49 | 4505 | 2.71 | 0.22 | <0.001 |
|  | Prefers gestures ^c^ | 2 | 8637 | 1.59 | 0.55 | 4501 | 1.57 | 0.24 | <0.001 |
|  | Pronouncing certain sounds ^c^ | 1 | 8587 | 0.80 | 0.40 | 4551 | 0.79 | 0.17 | 0.353 |
| 81m | Stumbles on words ^c^ | 3 | 8429 | 2.81 | 0.44 | 4709 | 2.77 | 0.20 | <0.001 |
|  | Prefers gestures ^c^ | 2 | 8418 | 1.67 | 0.51 | 4720 | 1.63 | 0.23 | <0.001 |
|  | Pronouncing certain sounds ^c^ | 1 | 8400 | 0.86 | 0.34 | 4738 | 0.85 | 0.15 | 0.003 |
| 38m | EAS – Sociability | 20 | 10061 | 13.20 | 3.11 | 3077 | 13.12 | 1.09 | 0.042 |
|  | Stays mainly silent ^c^ | 2 | 9616 | 1.90 | 0.32 | 3522 | 1.87 | 0.14 | <0.001 |
|  | Avoids eye contact ^c^ | 2 | 9676 | 1.80 | 0.45 | 3462 | 1.76 | 0.15 | <0.001 |
| 57m | EAS – Sociability | 20 | 9473 | 13.19 | 2.61 | 3665 | 13.18 | 1.05 | 0.621 |
|  | Stays mainly silent ^c^ | 2 | 8937 | 1.82 | 0.39 | 4201 | 1.81 | 0.15 | 0.039 |
|  | Avoids eye contact ^c^ | 2 | 9034 | 1.73 | 0.48 | 4104 | 1.71 | 0.17 | <0.001 |
| 69m | EAS – Sociability | 20 | 8637 | 13.25 | 2.62 | 4501 | 13.27 | 1.14 | 0.653 |
|  | Stays mainly silent ^c^ | 2 | 8061 | 1.80 | 0.41 | 5077 | 1.80 | 0.16 | 0.988 |
|  | Avoids eye contact ^c^ | 2 | 8165 | 1.73 | 0.49 | 4973 | 1.71 | 0.19 | 0.013 |
| 81m | Stays mainly silent ^c^ | 2 | 8043 | 1.79 | 0.41 | 5095 | 1.79 | 0.15 | 0.618 |
|  | Avoids eye contact ^c^ | 2 | 8126 | 1.76 | 0.47 | 5012 | 1.74 | 0.19 | 0.002 |
| 91m | DAWBA – Social fears ^c^ | 12 | 8179 | 11.11 | 1.61 | 4959 | 11.02 | 0.51 | <0.001 |

^a^ The observed maximum scores shown above were equivalent to the maximum feasible scores for all traits except *WISC – Verbal IQ*. For most traits, the minimum feasible score of zero was also observed.

^b^ Using t-test with unequal variances.

###### ^c^ Scores have been reversed so that high scores reflected a favourable response.
