# Supplementary material for: Traits Contributing to the Autistic Spectrum
Source: PLoS One. 2010 Sep 8;5(9):e12633. doi: 10.1371/journal.pone.0012633 (PMC2935882; doi:10.1371/journal.pone.0012633)
Supplement: Table S3 — Maximum and estimated communalities for individual measures using different data sets. (0.19 MB DOC) [file pone.0012633.s007.doc]

Table S3: Maximum (M) and estimated (E) communalities for individual measures using different data sets

| Age | Trait | Standard | | Pairwise | | Complete | | Low IQ | | High IQ | | Single | |
| --- | --- | --- | --- | --- | --- | --- | --- | --- | --- | --- | --- | --- | --- |
|  |  | M | E | M | E | M | E | M | E | M | E | M | E |
| 6m | DDST – Communication | 0.19 | 0.14 | 0.17 | 0.12 | 0.16 | 0.11 | 0.20 | 0.12 | 0.20 | 0.12 |  |  |
|  | Pretend play | 0.16 | 0.09 | 0.14 | 0.07 | 0.16 | 0.08 | 0.24 | 0.13 | 0.19 | 0.10 |  |  |
| 15m | CDI – understand score | 0.48 | 0.40 | 0.45 | 0.38 | 0.47 | 0.37 | 0.49 | 0.39 | 0.47 | 0.38 | 0.47 | 0.47 |
|  | CDI – Vocabulary | 0.65 | 0.62 | 0.62 | 0.59 | 0.66 | 0.61 | 0.66 | 0.60 | 0.67 | 0.64 | 0.65 | 0.66 |
|  | CDI – response to language | 0.12 | 0.07 | 0.10 | 0.06 | 0.10 | 0.04 | 0.14 | 0.06 | 0.13 | 0.05 | 0.10 | 0.09 |
|  | CDI – imitates words | 0.38 | 0.34 | 0.35 | 0.32 | 0.37 | 0.31 | 0.38 | 0.33 | 0.40 | 0.34 | 0.37 | 0.35 |
|  | CDI – gestures | 0.43 | 0.39 | 0.40 | 0.36 | 0.38 | 0.33 | 0.45 | 0.38 | 0.36 | 0.31 | 0.42 | 0.45 |
|  | CDI – objects | 0.49 | 0.45 | 0.46 | 0.42 | 0.47 | 0.40 | 0.52 | 0.43 | 0.45 | 0.40 | 0.47 | 0.49 |
| 18m | DDST – communication | 0.62 | 0.62 | 0.58 | 0.59 | 0.61 | 0.60 | 0.61 | 0.60 | 0.62 | 0.60 | 0.62 | 0.64 |
|  | Pretend play | 0.22 | 0.18 | 0.19 | 0.16 | 0.17 | 0.13 | 0.27 | 0.19 | 0.18 | 0.14 |  |  |
| 24m | CDI – Vocabulary | 0.82 | 0.78 | 0.79 | 0.75 | 0.81 | 0.76 | 0.83 | 0.77 | 0.79 | 0.76 | 0.81 | 0.82 |
|  | CDI – grammar (regular) | 0.69 | 0.61 | 0.66 | 0.58 | 0.66 | 0.56 | 0.68 | 0.59 | 0.67 | 0.58 | 0.67 | 0.70 |
|  | CDI – grammar (irregular) | 0.72 | 0.62 | 0.69 | 0.58 | 0.69 | 0.59 | 0.73 | 0.62 | 0.69 | 0.61 | 0.70 | 0.67 |
|  | CDI – combines words | 0.59 | 0.51 | 0.55 | 0.47 | 0.55 | 0.45 | 0.59 | 0.47 | 0.52 | 0.40 | 0.57 | 0.55 |
| 30m | Pretend play | 0.50 | 0.32 | 0.43 | 0.28 | 0.47 | 0.27 | 0.52 | 0.32 | 0.48 | 0.27 |  |  |
| 38m | CDI – Vocabulary | 0.68 | 0.55 | 0.64 | 0.53 | 0.62 | 0.52 | 0.69 | 0.57 | 0.45 | 0.38 |  |  |
|  | CDI – grammar (regular) | 0.61 | 0.54 | 0.55 | 0.50 | 0.53 | 0.48 | 0.64 | 0.56 | 0.46 | 0.45 |  |  |
|  | CDI – grammar (irregular) | 0.59 | 0.43 | 0.56 | 0.40 | 0.54 | 0.38 | 0.64 | 0.47 | 0.47 | 0.36 |  |  |
|  | CDI – complexity | 0.55 | 0.48 | 0.49 | 0.43 | 0.45 | 0.39 | 0.56 | 0.49 | 0.38 | 0.33 |  |  |
|  | CDI – combines words | 0.60 | 0.57 | 0.53 | 0.50 | 0.40 | 0.33 | 0.55 | 0.51 | 0.25 | 0.21 |  |  |
|  | Communication | 0.54 | 0.51 | 0.47 | 0.44 | 0.43 | 0.39 | 0.55 | 0.49 | 0.34 | 0.30 |  |  |
|  | Intelligibility | 0.44 | 0.40 | 0.36 | 0.33 | 0.39 | 0.33 | 0.51 | 0.46 | 0.30 | 0.26 |  |  |
| 42m | Pretend play | 0.52 | 0.34 | 0.45 | 0.30 | 0.46 | 0.27 | 0.56 | 0.37 | 0.47 | 0.26 | 0.29 | 0.29 |
| 57m | Communication | 0.77 | 0.74 | 0.65 | 0.59 | 0.57 | 0.55 | 0.74 | 0.69 | 0.42 | 0.37 | 0.66 | 0.69 |
|  | Musical | 0.50 | 0.37 | 0.42 | 0.28 | 0.38 | 0.24 | 0.53 | 0.38 | 0.40 | 0.29 | 0.38 | 0.35 |
|  | Intelligibility | 0.17 | 0.14 | 0.13 | 0.10 | 0.11 | 0.07 | 0.23 | 0.18 | 0.13 | 0.06 | 0.12 | 0.13 |
|  | Combines words | 0.64 | 0.59 | 0.53 | 0.48 | 0.27 | 0.20 | 0.58 | 0.47 | 0.22 | 0.12 | 0.49 | 0.50 |
| 69m | Communication | 0.80 | 0.75 | 0.68 | 0.60 | 0.63 | 0.59 | 0.76 | 0.68 | 0.38 | 0.28 |  |  |
|  | Musical | 0.55 | 0.38 | 0.46 | 0.29 | 0.44 | 0.26 | 0.57 | 0.37 | 0.42 | 0.23 |  |  |
|  | Intelligibility | 0.13 | 0.08 | 0.09 | 0.05 | 0.09 | 0.04 | 0.17 | 0.10 | 0.11 | 0.02 |  |  |
|  | Combines words | 0.66 | 0.59 | 0.55 | 0.47 | 0.23 | 0.16 | 0.55 | 0.42 | 0.16 | 0.07 |  |  |
| 81m | Communication | 0.79 | 0.74 | 0.66 | 0.56 | 0.56 | 0.49 | 0.72 | 0.63 | 0.27 | 0.19 |  |  |
|  | Musical | 0.51 | 0.35 | 0.41 | 0.25 | 0.37 | 0.21 | 0.52 | 0.34 | 0.35 | 0.18 |  |  |
|  | Intelligibility | 0.12 | 0.08 | 0.08 | 0.05 | 0.10 | 0.04 | 0.18 | 0.10 | 0.10 | 0.03 |  |  |
|  | Combines words | 0.55 | 0.49 | 0.38 | 0.30 | 0.22 | 0.15 | 0.47 | 0.36 | 0.09 | 0.02 |  |  |
| 9y | CCC – intelligibility & fluency | 0.63 | 0.59 | 0.50 | 0.42 | 0.43 | 0.36 | 0.65 | 0.55 | 0.33 | 0.24 | 0.61 | 0.65 |
|  | CCC – syntax score | 0.67 | 0.63 | 0.48 | 0.41 | 0.31 | 0.24 | 0.59 | 0.47 | 0.17 | 0.08 | 0.61 | 0.64 |
|  | CCC – coherence | 0.68 | 0.62 | 0.57 | 0.49 | 0.50 | 0.40 | 0.68 | 0.56 | 0.37 | 0.27 | 0.66 | 0.68 |
| 42m | Rutter Prosocial | 0.51 | 0.47 | 0.45 | 0.40 | 0.44 | 0.38 | 0.54 | 0.46 | 0.48 | 0.43 |  |  |
| 47m | SDQ Prosocial | 0.50 | 0.45 | 0.43 | 0.38 | 0.46 | 0.39 | 0.52 | 0.43 | 0.47 | 0.37 |  |  |
| 57m | Empathy | 0.48 | 0.44 | 0.41 | 0.36 | 0.39 | 0.31 | 0.54 | 0.46 | 0.41 | 0.35 |  |  |
| 69m | Empathy | 0.54 | 0.48 | 0.45 | 0.40 | 0.44 | 0.38 | 0.58 | 0.51 | 0.42 | 0.34 |  |  |
| 81m | Empathy | 0.55 | 0.50 | 0.47 | 0.42 | 0.44 | 0.39 | 0.59 | 0.49 | 0.47 | 0.41 | 0.42 | 0.41 |
|  | SDQ Prosocial | 0.61 | 0.60 | 0.52 | 0.51 | 0.53 | 0.50 | 0.60 | 0.56 | 0.59 | 0.55 | 0.39 | 0.40 |
| 91m | SCDC | 0.55 | 0.53 | 0.44 | 0.44 | 0.39 | 0.37 | 0.56 | 0.52 | 0.42 | 0.38 | 0.52 | 0.54 |
| 97m | SDQ Prosocial | 0.61 | 0.59 | 0.50 | 0.48 | 0.54 | 0.51 | 0.60 | 0.54 | 0.60 | 0.56 |  |  |
| 9y | SDQ Prosocial | 0.57 | 0.55 | 0.47 | 0.46 | 0.49 | 0.45 | 0.59 | 0.55 | 0.54 | 0.50 |  |  |
|  | CCC – conversational rapport | 0.57 | 0.51 | 0.46 | 0.40 | 0.43 | 0.35 | 0.55 | 0.47 | 0.44 | 0.36 | 0.54 | 0.56 |
| 38m | Echoes what said | 0.24 | 0.21 | 0.19 | 0.16 | 0.22 | 0.18 | 0.26 | 0.21 | 0.22 | 0.16 |  |  |
| 57m | Echoes what said | 0.37 | 0.30 | 0.28 | 0.21 | 0.31 | 0.21 | 0.38 | 0.32 | 0.29 | 0.18 |  |  |
| 69m | Echoes what said | 0.41 | 0.33 | 0.30 | 0.22 | 0.26 | 0.18 | 0.41 | 0.36 | 0.33 | 0.21 |  |  |
| 81m | Echoes what said | 0.35 | 0.30 | 0.26 | 0.21 | 0.25 | 0.19 | 0.36 | 0.32 | 0.25 | 0.14 | 0.23 | 0.21 |
|  | Nonverbal communication | 0.20 | 0.18 | 0.13 | 0.12 | 0.15 | 0.10 | 0.21 | 0.14 | 0.15 | 0.10 | 0.18 | 0.19 |
| 8y | WOLD – comprehension | 0.34 | 0.23 | 0.26 | 0.18 | 0.27 | 0.15 | 0.24 | 0.07 | 0.17 | 0.05 | 0.32 | 0.36 |
|  | WOLD – oral expression | 0.50 | 0.43 | 0.37 | 0.30 | 0.39 | 0.27 | 0.28 | 0.14 | 0.18 | 0.10 | 0.48 | 0.54 |
|  | Nonword repetition | 0.42 | 0.37 | 0.28 | 0.24 | 0.27 | 0.20 | 0.29 | 0.21 | 0.21 | 0.14 | 0.40 | 0.42 |
|  | WISC – verbal IQ | 0.57 | 0.51 | 0.44 | 0.39 | 0.46 | 0.35 | 0.31 | 0.18 | 0.23 | 0.11 | 0.56 | 0.64 |
|  | DANVA – faces | 0.20 | 0.13 | 0.10 | 0.06 | 0.11 | 0.05 | 0.16 | 0.06 | 0.12 | 0.04 | 0.17 | 0.15 |
| 9y | CCC – inappropriate initiation | 0.44 | 0.37 | 0.37 | 0.29 | 0.37 | 0.26 | 0.49 | 0.38 | 0.36 | 0.26 | 0.42 | 0.50 |
|  | CCC – stereotyped conversation | 0.49 | 0.42 | 0.42 | 0.33 | 0.39 | 0.26 | 0.51 | 0.42 | 0.38 | 0.30 | 0.47 | 0.51 |
|  | CCC – conversational context | 0.64 | 0.59 | 0.55 | 0.49 | 0.48 | 0.38 | 0.64 | 0.56 | 0.43 | 0.36 | 0.63 | 0.67 |
| 18m | Repetitive behaviour | 0.18 | 0.14 | 0.13 | 0.09 | 0.12 | 0.06 | 0.21 | 0.13 | 0.14 | 0.06 |  |  |
| 30m | Repetitive behaviour | 0.29 | 0.22 | 0.23 | 0.14 | 0.20 | 0.10 | 0.33 | 0.23 | 0.16 | 0.03 |  |  |
| 42m | Repetitive behaviour | 0.32 | 0.26 | 0.26 | 0.19 | 0.24 | 0.15 | 0.34 | 0.25 | 0.14 | 0.03 |  |  |
| 57m | Repetitive behaviour | 0.39 | 0.35 | 0.32 | 0.24 | 0.28 | 0.18 | 0.36 | 0.31 | 0.24 | 0.10 |  |  |
| 69m | Repetitive behaviour | 0.41 | 0.39 | 0.33 | 0.26 | 0.22 | 0.14 | 0.44 | 0.38 | 0.22 | 0.09 |  |  |
| 77m | Repetitive behaviour | 0.37 | 0.35 | 0.28 | 0.23 | 0.23 | 0.14 | 0.48 | 0.41 | 0.25 | 0.13 | 0.23 | 0.18 |
| 91m | DAWBA – Number compulsions | 0.93 | 0.53 | 0.92 | 0.64 | 0.91 | 0.74 | 0.95 | 0.59 | 0.90 | 0.81 | 0.93 | 0.93 |
|  | DAWBA – Compulsions score | 0.93 | 0.59 | 0.92 | 0.69 | 0.91 | 0.79 | 0.95 | 0.64 | 0.90 | 0.83 | 0.93 | 0.95 |
|  | DAWBA – Tics or twitches | 0.13 | 0.09 | 0.09 | 0.06 | 0.11 | 0.05 | 0.21 | 0.12 | 0.16 | 0.07 | 0.11 | 0.06 |
| 38m | Stumbles on words | 0.19 | 0.13 | 0.16 | 0.11 | 0.19 | 0.12 | 0.20 | 0.14 | 0.22 | 0.13 |  |  |
|  | Prefers gestures | 0.33 | 0.27 | 0.27 | 0.22 | 0.26 | 0.20 | 0.38 | 0.28 | 0.23 | 0.16 |  |  |
| 57m | Stumbles on words | 0.38 | 0.28 | 0.32 | 0.23 | 0.34 | 0.24 | 0.39 | 0.28 | 0.36 | 0.25 |  |  |
|  | Prefers gestures | 0.39 | 0.29 | 0.33 | 0.24 | 0.33 | 0.24 | 0.43 | 0.29 | 0.34 | 0.21 |  |  |
|  | Pronouncing certain sounds | 0.37 | 0.34 | 0.31 | 0.29 | 0.33 | 0.26 | 0.41 | 0.35 | 0.35 | 0.26 |  |  |
| 69m | Stumbles on words | 0.43 | 0.32 | 0.36 | 0.27 | 0.36 | 0.28 | 0.50 | 0.40 | 0.35 | 0.23 |  |  |
|  | Prefers gestures | 0.41 | 0.30 | 0.34 | 0.24 | 0.35 | 0.26 | 0.43 | 0.30 | 0.38 | 0.23 |  |  |
|  | Pronouncing certain sounds | 0.46 | 0.41 | 0.40 | 0.35 | 0.41 | 0.34 | 0.52 | 0.48 | 0.46 | 0.37 |  |  |
| 81m | Stumbles on words | 0.40 | 0.30 | 0.31 | 0.24 | 0.34 | 0.27 | 0.47 | 0.38 | 0.29 | 0.19 | 0.23 | 0.23 |
|  | Prefers gestures | 0.38 | 0.28 | 0.30 | 0.21 | 0.30 | 0.22 | 0.39 | 0.28 | 0.30 | 0.17 | 0.21 | 0.21 |
|  | Pronouncing certain sounds | 0.43 | 0.38 | 0.35 | 0.31 | 0.36 | 0.30 | 0.49 | 0.44 | 0.42 | 0.33 | 0.22 | 0.21 |
| 38m | EAS – Sociability | 0.38 | 0.30 | 0.33 | 0.27 | 0.38 | 0.31 | 0.37 | 0.25 | 0.38 | 0.26 |  |  |
|  | Stays mainly silent | 0.30 | 0.26 | 0.23 | 0.20 | 0.18 | 0.15 | 0.31 | 0.23 | 0.22 | 0.15 |  |  |
|  | Avoids eye contact | 0.21 | 0.16 | 0.16 | 0.12 | 0.16 | 0.09 | 0.26 | 0.17 | 0.19 | 0.10 |  |  |
| 57m | EAS – Sociability | 0.49 | 0.38 | 0.43 | 0.36 | 0.46 | 0.38 | 0.49 | 0.32 | 0.49 | 0.35 |  |  |
|  | Stays mainly silent | 0.36 | 0.32 | 0.29 | 0.27 | 0.27 | 0.23 | 0.33 | 0.28 | 0.36 | 0.30 |  |  |
|  | Avoids eye contact | 0.29 | 0.23 | 0.23 | 0.18 | 0.22 | 0.15 | 0.35 | 0.27 | 0.27 | 0.18 |  |  |
| 69m | EAS – Sociability | 0.47 | 0.37 | 0.41 | 0.34 | 0.44 | 0.35 | 0.49 | 0.33 | 0.45 | 0.31 | 0.12 | 0.13 |
|  | Stays mainly silent | 0.37 | 0.34 | 0.29 | 0.27 | 0.31 | 0.29 | 0.39 | 0.35 | 0.36 | 0.30 |  |  |
|  | Avoids eye contact | 0.35 | 0.26 | 0.27 | 0.21 | 0.25 | 0.16 | 0.42 | 0.26 | 0.29 | 0.19 |  |  |
| 81m | Stays mainly silent | 0.33 | 0.29 | 0.26 | 0.23 | 0.28 | 0.24 | 0.34 | 0.27 | 0.34 | 0.31 | 0.16 | 0.18 |
|  | Avoids eye contact | 0.34 | 0.26 | 0.26 | 0.20 | 0.26 | 0.19 | 0.38 | 0.25 | 0.30 | 0.21 | 0.22 | 0.25 |
| 91m | DAWBA – Social fears | 0.20 | 0.16 | 0.13 | 0.10 | 0.16 | 0.12 | 0.24 | 0.17 | 0.18 | 0.11 | 0.18 | 0.21 |
|  | Total communalities | 42.7 | 36.1 | 36.0 | 29.9 | 34.4 | 27.3 | 43.4 | 34.4 | 32.6 | 24.5 | 18.9 | 19.5 |
|  | N | 13138 | | 7811 | | 2481 | | 1816 | | 1863 | | 13138 | |

The ‘maximum’ communalities were estimated by the R^2^ from linear regression analyses of each variable on the remaining 92 variables. The estimated communalities were derived from factor analyses retaining 7 factors. Estimated communalities exceeded the ‘maximum’ for the *Single* data set.

The number of observations for the factor analysis of pairwise correlations varied between 5201 and 11316 with an average of 7811.

Data sets:

Standard The main data set used in this study based upon imputed data derived from 93 variables.

Imputed Imputation based upon 93 variables, gender, family adversity (during pregnancy, first 2 years after birth, 3^rd^ and 4^th^ years), parenting score (6m). Results not shown being very similar to standard data set.

Pairwise Loadings derived from pairwise correlations

Complete Only observed data for all 93 variables (N=2481)

Low IQ Analysis restricted to bottom quartile of FSIQ (N=1816)

High IQ Analysis restricted to top quartile of FSIQ (N=1863)

Single Standard data set but restricted to 44 variables excluding repeat measures
