# Supplementary material for: Traits Contributing to the Autistic Spectrum
Source: PLoS One. 2010 Sep 8;5(9):e12633. doi: 10.1371/journal.pone.0012633 (PMC2935882; doi:10.1371/journal.pone.0012633)
Supplement: Table S4 — Comparisons of factor loadings generated for different data sets compared to the results for the standard imputed data set. (0.05 MB DOC) [file pone.0012633.s008.doc]

Table S4: Comparisons of factor loadings generated for different data sets compared to the results for the standard imputed data set (as shown in Table 2)

| Factor | Variables | | Data sets | | | | | | |
| --- | --- | --- | --- | --- | --- | --- | --- | --- | --- |
|  | A | B | Imputed | Pairwise | Complete | Low IQ | High IQ | Oblique | Single |
| 1 | 23 | 8 |  |  |  |  | 8 |  | 1 |
| 2 | 23 | 12 |  | 1 | 1 | 2 | 5 | 4 | 1 |
| 3 | 10 | 4 |  |  |  |  |  |  | 1 |
| 4 | 13 | 10 | 1 |  |  | 4 | 4 |  | 4 |
| 5 | 9 | 4 |  | 1 | 1 | 2 | 5 |  | 1 |
| 6 | 11 | 3 |  | 2 | 3 | 4 | 5 | 1 | 3 |
| 7 | 12 | 4 |  |  |  | 2 |  |  | 1 |
| 1 | 70 | 36 |  | 4 | 4 | 3 | 7 | 1 | 7 |
| 2 | 70 | 32 |  |  |  | 1 |  |  | 1 |
| 3 | 83 | 40 |  | 1 | 2 | 3 | 2 | 2 | 4 |
| 4 | 80 | 34 |  | 1 | 5 | 11 | 7 | 5 | 3 |
| 5 | 84 | 40 |  | 2 | 3 | 1 | 3 | 3 | 2 |
| 6 | 82 | 41 |  |  |  | 2 | 3 |  | 9 |
| 7 | 81 | 40 |  | 1 | 1 |  |  | 1 | 2 |
| Discrepancy rate (%) | | | 0.15 | 2.00 | 3.07 | 5.38 | 7.53 | 2.61 | 12.99 |

The table shows the frequencies of discrepancies between factor loadings (recoded into 0/1 based upon a criterion of 0.3) for loadings associated primarily with each of the seven factors (top half) and for other loadings (bottom half) compared to the results from the standard imputed data set based upon the 93 variables. The other data sets are:

Variable set A (93 variables):

Imputed Imputation based upon 93 variables, gender, family adversity (during pregnancy, first 2 years after birth, 3^rd^ and 4^th^ years), parenting score (6m)

Pairwise Loadings derived from pairwise correlations

Complete Only observed data for all 93 variables (N=2481)

Low IQ Analysis restricted to bottom quartile of FSIQ (N=1816)

High IQ Analysis restricted to top quartile of FSIQ (N=1863)

Oblique Standard imputed data but using promax rotation

Variable set B (44 variables – excluding repeat measures):

Single Observations from Standard imputed dataset

All factor loadings, except *Oblique*, were transformed using varimax rotation. Full-scale IQ at 8y was only available for 7354 children. Factors 1 and 2 shared 8 or 1 variables (sets A or B) hence the total number of variables associated with the factors (top half of the table) is 101 or 45.

Factors: 1 *Verbal ability*, 2 *Language acquisition*, 3 *Social understanding*, 4 *Semantic-pragmatic skills*, 5 *Repetitive Behaviour*, 6 *Articulation* and 7 *Social inhibition*
