# Supplementary material for: Traits Contributing to the Autistic Spectrum
Source: PLoS One. 2010 Sep 8;5(9):e12633. doi: 10.1371/journal.pone.0012633 (PMC2935882; doi:10.1371/journal.pone.0012633)
Supplement: Table S5 — Factors analyses of individual measures classified by age. (0.17 MB DOC) [file pone.0012633.s009.doc]

Table S5: Factor analyses of 8 traits (6-15m), 22 traits (18-38m), 31 traits (42-77m) and 32 traits (81m-9y) after varimax rotation (N = 13,138)

(A) 6-15m

| Age | Trait | Factor 1 |
| --- | --- | --- |
| 6m | DDST – Communication | **0.39** |
|  | Pretend play | **0.31** |
| 15m | CDI – understand score | **0.70** |
|  | CDI – Vocabulary | **0.79** |
|  | CDI – response to language | 0.25 |
|  | CDI – imitates words | **0.57** |
|  | CDI – gestures | **0.64** |
|  | CDI – objects | **0.70** |
|  | Variance explained | 107.8% |

(B) 18-38m

| Age | Trait | Factor Loadings | | | | |
| --- | --- | --- | --- | --- | --- | --- |
|  |  | 1 | 2 | 3 | 4 | 5 |
| 18m | DDST – communication | 0.18 | **0.70** | 0.17 | 0.11 | 0.05 |
|  | Pretend play | 0.12 | **0.31** | 0.19 | -0.15 | 0.15 |
| 24m | CDI – Vocabulary | **0.33** | **0.83** | 0.05 | 0.22 | 0.04 |
|  | CDI – grammar (regular) | 0.23 | **0.73** | 0.08 | **0.34** | -0.07 |
|  | CDI – grammar (irregular) | 0.21 | **0.81** | -0.05 | 0.14 | 0.00 |
|  | CDI – combines words | 0.31 | **0.57** | 0.18 | 0.35 | -0.04 |
| 30m | Pretend play | 0.14 | **0.39** | 0.18 | -0.06 | 0.14 |
| 38m | CDI – Vocabulary | **0.77** | 0.27 | 0.15 | -0.05 | 0.15 |
|  | CDI – grammar (regular) | **0.69** | **0.31** | 0.16 | 0.17 | 0.06 |
|  | CDI – grammar (irregular) | **0.68** | **0.38** | 0.02 | -0.06 | 0.09 |
|  | CDI – complexity | **0.68** | 0.22 | 0.17 | 0.10 | 0.08 |
|  | CDI – combines words | **0.64** | 0.10 | 0.33 | 0.03 | 0.07 |
|  | Communication | **0.56** | 0.22 | 0.27 | 0.29 | 0.17 |
|  | Intelligibility | **0.53** | 0.16 | 0.29 | 0.21 | 0.05 |
| 38m | Echoes what said | 0.12 | 0.13 | 0.00 | 0.29 | 0.14 |
| 18m | Repetitive behaviour | 0.06 | 0.03 | 0.06 | 0.14 | **0.44** |
| 30m | Repetitive behaviour | 0.08 | 0.01 | 0.06 | 0.09 | **0.43** |
| 38m | Stumbles on words | -0.01 | 0.04 | 0.04 | 0.21 | 0.08 |
|  | Prefers gestures | **0.33** | 0.16 | 0.16 | **0.33** | 0.09 |
| 38m | EAS – Sociability | 0.08 | 0.08 | **0.30** | 0.01 | 0.02 |
|  | Stays mainly silent | 0.27 | 0.09 | **0.42** | 0.05 | 0.05 |
|  | Avoids eye contact | 0.14 | 0.02 | **0.31** | 0.14 | 0.10 |
|  | Variance explained | 43.1% | 41.3% | 10.5% | 9.7% | 6.6% |

Total variance explained by 5 factors = 111.3%

Table S5: continued

(C) 42-77m

| Age | Trait | Factor Loadings | | | | | |
| --- | --- | --- | --- | --- | --- | --- | --- |
|  |  | 1 | 2 | 3 | 4 | 5 | 6 |
| 42m | Pretend play | 0.23 | **0.40** | 0.05 | 0.05 | 0.10 | 0.05 |
| 57m | Communication | **0.76** | 0.23 | 0.18 | 0.18 | 0.11 | 0.21 |
|  | Musical | **0.51** | 0.32 | 0.13 | 0.00 | 0.07 | 0.14 |
|  | Intelligibility | 0.23 | 0.06 | 0.10 | 0.05 | 0.08 | 0.25 |
|  | Combines words | **0.72** | 0.09 | 0.20 | 0.14 | 0.11 | 0.02 |
| 69m | Communication | **0.75** | 0.21 | 0.16 | 0.25 | 0.09 | 0.18 |
|  | Musical | **0.53** | **0.30** | 0.11 | 0.07 | 0.07 | 0.10 |
|  | Intelligibility | 0.20 | 0.04 | 0.08 | 0.05 | 0.07 | 0.15 |
|  | Combines words | **0.72** | 0.08 | 0.14 | 0.18 | 0.10 | 0.00 |
| 42m | Rutter Prosocial | 0.10 | **0.68** | 0.07 | 0.05 | 0.14 | 0.06 |
| 47m | SDQ Prosocial | 0.08 | **0.67** | 0.09 | 0.04 | 0.13 | 0.05 |
| 57m | Empathy | 0.21 | **0.61** | 0.16 | 0.15 | 0.01 | 0.06 |
| 69m | Empathy | 0.22 | **0.56** | 0.17 | 0.21 | 0.01 | 0.04 |
| 57m | Echoes what said | 0.11 | 0.05 | **0.53** | 0.07 | -0.01 | -0.06 |
| 69m | Echoes what said | 0.18 | 0.05 | **0.53** | 0.09 | 0.01 | -0.07 |
| 42m | Repetitive behaviour | 0.11 | 0.08 | 0.06 | **0.54** | 0.04 | 0.03 |
| 57m | Repetitive behaviour | 0.12 | 0.09 | 0.09 | **0.65** | 0.06 | 0.04 |
| 69m | Repetitive behaviour | 0.14 | 0.09 | 0.11 | **0.66** | 0.04 | 0.05 |
| 77m | Repetitive behaviour | 0.11 | 0.09 | 0.09 | **0.59** | 0.05 | 0.07 |
| 57m | Stumbles on words | 0.05 | 0.10 | **0.45** | 0.07 | 0.05 | 0.26 |
|  | Prefers gestures | 0.16 | 0.08 | **0.45** | 0.04 | 0.13 | 0.23 |
|  | Pronouncing certain sounds | 0.08 | 0.05 | 0.07 | 0.04 | 0.04 | **0.62** |
| 69m | Stumbles on words | 0.09 | 0.11 | **0.44** | 0.11 | 0.05 | 0.29 |
|  | Prefers gestures | 0.19 | 0.09 | **0.45** | 0.05 | 0.11 | 0.23 |
|  | Pronouncing certain sounds | 0.14 | 0.05 | 0.09 | 0.05 | 0.05 | **0.63** |
| 57m | EAS – Sociability | 0.04 | 0.10 | -0.01 | -0.01 | **0.65** | 0.03 |
|  | Stays mainly silent | 0.14 | 0.03 | 0.12 | 0.05 | **0.48** | 0.07 |
|  | Avoids eye contact | 0.05 | 0.16 | 0.30 | 0.08 | **0.31** | -0.02 |
| 69m | EAS – Sociability | 0.02 | 0.10 | -0.03 | 0.01 | **0.65** | 0.04 |
|  | Stays mainly silent | 0.12 | 0.00 | 0.10 | 0.09 | **0.49** | 0.08 |
|  | Avoids eye contact | 0.07 | 0.16 | 0.32 | 0.09 | **0.30** | 0.00 |
|  | Variance explained | 27.8% | 19.3% | 16.1% | 15.4% | 14.4% | 10.9% |

Total variance explained by 6 factors = 103.9%

Table S5: continued

(D) 81m-9y

| Age | Trait | Factor Loadings | | | | | | |
| --- | --- | --- | --- | --- | --- | --- | --- | --- |
|  |  | 1 | 2 | 3 | 4 | 5 | 6 | 7 |
| 81m | Communication | 0.19 | 0.25 | 0.18 | **0.67** | 0.19 | **0.30** | 0.15 |
|  | Musical | 0.22 | 0.18 | 0.12 | **0.39** | 0.09 | 0.19 | 0.20 |
|  | Intelligibility | 0.06 | 0.08 | 0.05 | 0.14 | 0.05 | 0.20 | 0.10 |
|  | Combines words | 0.08 | 0.15 | 0.05 | **0.63** | 0.10 | 0.13 | 0.09 |
| 9y | CCC – intelligibility & fluency | 0.10 | 0.25 | 0.10 | **0.45** | 0.07 | 0.59 | 0.19 |
|  | CCC – syntax score | 0.11 | 0.25 | 0.18 | **0.68** | 0.13 | 0.29 | 0.15 |
|  | CCC – coherence | 0.25 | 0.25 | 0.35 | **0.44** | 0.10 | **0.40** | 0.28 |
| 81m | Empathy | **0.56** | 0.11 | 0.22 | 0.19 | 0.10 | 0.11 | 0.14 |
|  | SDQ Prosocial | **0.79** | 0.06 | 0.04 | 0.07 | 0.04 | 0.08 | 0.11 |
| 91m | SCDC | **0.50** | 0.12 | **0.45** | 0.15 | 0.16 | 0.13 | 0.15 |
| 97m | SDQ Prosocial | **0.78** | 0.03 | 0.06 | 0.07 | 0.04 | 0.05 | 0.12 |
| 9y | SDQ Prosocial | **0.76** | -0.02 | 0.03 | 0.07 | 0.05 | 0.06 | 0.14 |
|  | CCC – conversational rapport | **0.38** | 0.15 | 0.28 | 0.24 | 0.11 | 0.17 | **0.48** |
| 81m | Echoes what said | 0.03 | 0.22 | **0.35** | 0.11 | 0.09 | 0.10 | 0.17 |
|  | Nonverbal communication | 0.15 | 0.19 | 0.27 | 0.12 | 0.04 | 0.06 | 0.22 |
| 8y | WOLD – comprehension | 0.05 | **0.59** | 0.09 | 0.07 | 0.06 | 0.02 | 0.04 |
|  | WOLD – oral expression | 0.06 | **0.70** | 0.12 | 0.15 | 0.06 | 0.12 | 0.05 |
|  | Nonword repetition | -0.01 | **0.52** | 0.13 | 0.15 | 0.01 | 0.27 | 0.03 |
|  | WISC – verbal IQ | 0.02 | **0.74** | 0.19 | 0.18 | 0.06 | 0.07 | 0.10 |
|  | DANVA – faces | 0.11 | 0.25 | 0.09 | 0.14 | 0.04 | 0.11 | 0.15 |
| 9y | CCC – inappropriate initiation | 0.06 | 0.12 | **0.67** | 0.04 | 0.07 | 0.08 | -0.15 |
|  | CCC – stereotyped conversation | 0.08 | 0.10 | **0.69** | 0.05 | 0.12 | 0.09 | 0.10 |
|  | CCC – conversational context | 0.21 | 0.27 | **0.65** | 0.23 | 0.13 | 0.16 | 0.21 |
| 91m | DAWBA – Number compulsions | 0.10 | 0.09 | 0.17 | 0.14 | **0.92** | 0.07 | 0.13 |
|  | DAWBA – Compulsions score | 0.11 | 0.09 | 0.18 | 0.18 | **0.92** | 0.08 | 0.12 |
|  | DAWBA – Tics or twitches | 0.09 | 0.03 | 0.07 | 0.10 | 0.11 | 0.07 | 0.05 |
| 81m | Stumbles on words | 0.07 | 0.14 | 0.19 | 0.11 | 0.03 | **0.39** | 0.18 |
|  | Prefers gestures | 0.08 | 0.15 | 0.15 | 0.15 | 0.05 | 0.28 | 0.25 |
|  | Pronouncing certain sounds | 0.06 | 0.04 | 0.04 | 0.12 | 0.03 | **0.49** | 0.06 |
| 81m | Stays mainly silent | 0.05 | -0.02 | -0.06 | 0.09 | 0.03 | 0.10 | **0.40** |
|  | Avoids eye contact | 0.19 | 0.05 | 0.14 | 0.06 | 0.04 | 0.11 | **0.45** |
| 91m | DAWBA – Social fears | 0.13 | 0.16 | 0.05 | 0.06 | 0.12 | 0.09 | **0.38** |
|  | Variance explained | 21.6% | 17.6% | 17.3% | 17.3% | 14.3% | 11.3% | 10.1% |

Total variance explained by 7 factors = 109.4%
