# Supplementary material for: Traits Contributing to the Autistic Spectrum
Source: PLoS One. 2010 Sep 8;5(9):e12633. doi: 10.1371/journal.pone.0012633 (PMC2935882; doi:10.1371/journal.pone.0012633)
Supplement: Table S6 — Prevalence of the worst decile calculated for each trait by ASD status and type of data. (0.18 MB DOC) [file pone.0012633.s010.doc]

Table S6: Prevalence (%) of the worst decile calculated for each trait by ASD status and type of data.

| Age | Trait | Observed Data | | All Data | | Rank |
| --- | --- | --- | --- | --- | --- | --- |
|  |  | Yes | No | Yes | No |  |
|  | 1: Verbal ability |  |  | 75.0 | 9.6 | 30 |
|  | 2: Language acquisition |  |  | 20.0 | 9.9 | 100 |
|  | 3: Social understanding |  |  | 63.8 | 9.7 | 15 |
|  | 4: Semantic-pragmatic skills |  |  | 42.5 | 9.8 | 74 |
|  | 5: Repetitive-stereotyped |  |  | 67.5 | 9.6 | 25 |
|  | 6: Articulation |  |  | 31.3 | 9.9 | 96 |
|  | 7: Social inhibition |  |  | 46.3 | 9.8 | 58 |
|  | Factor mean score |  |  | 88.8 | 9.5 | 1 |
| 6m | DDST – Communication | 13.2 | 12.3 | 11.3 | 10.6 | 97 |
|  | Pretend play | 41.2 | 26.9 | 35.0 | 23.2 | 98 |
| 15m | CDI – understand score | 29.7 | 8.8 | 30.0 | 7.6 | 86 |
|  | CDI – Vocabulary | 33.8 | 9.8 | 35.0 | 9.9 | 91 |
|  | CDI – response to language | 13.5 | 2.5 | 26.3 | 16.9 | 95 |
|  | CDI – imitates words | 21.6 | 7.4 | 22.5 | 6.6 | 90 |
|  | CDI – gestures | 39.2 | 9.2 | 38.8 | 7.9 | 71 |
|  | CDI – objects | 44.6 | 11.1 | 45.0 | 9.6 | 73 |
| 18m | DDST – communication | 39.2 | 8.2 | 47.5 | 10.8 | 63 |
|  | Pretend play | 32.4 | 11.8 | 31.3 | 10.0 | 92 |
| 24m | CDI – Vocabulary | 51.4 | 9.6 | 52.5 | 9.7 | 54 |
|  | CDI – grammar (regular) | 65.7 | 17.8 | 58.8 | 14.3 | 75 |
|  | CDI – grammar (irregular) | 48.6 | 8.9 | 53.8 | 11.2 | 79 |
|  | CDI – combines words | 59.4 | 17.6 | 51.3 | 13.6 | 70 |
| 30m | Pretend play | 52.9 | 11.4 | 52.5 | 9.5 | 59 |
| 38m | CDI – Vocabulary | 64.2 | 9.7 | 65.0 | 9.7 | 33 |
|  | CDI – grammar (regular) | 54.8 | 6.2 | 63.8 | 11.6 | 24 |
|  | CDI – grammar (irregular) | 61.2 | 9.5 | 62.5 | 9.7 | 36 |
|  | CDI – complexity | 47.8 | 9.8 | 56.3 | 10.0 | 50 |
|  | CDI – combines words | 56.9 | 7.1 | 62.5 | 9.7 | 39 |
|  | Communication | 66.2 | 6.7 | 66.3 | 6.5 | 10 |
|  | Intelligibility | 66.7 | 10.7 | 66.3 | 9.6 | 32 |
| 42m | Pretend play | 68.2 | 9.6 | 62.5 | 10.4 | 21 |
| 57m | Communication | 82.8 | 12.8 | 83.8 | 11.8 | 8 |
|  | Musical | 46.4 | 7.3 | 56.3 | 12.6 | 44 |
|  | Intelligibility | 46.7 | 9.9 | 46.3 | 7.8 | 81 |
|  | Combines words | 59.7 | 7.1 | 65.0 | 9.7 | 37 |
| 69m | Communication | 80.0 | 8.5 | 81.3 | 9.6 | 7 |
|  | Musical | 50.9 | 8.1 | 56.3 | 7.8 | 35 |
|  | Intelligibility | 31.0 | 9.6 | 51.3 | 10.1 | 83 |
|  | Combines words | 46.7 | 3.5 | 56.3 | 9.7 | 57 |
| 81m | Communication | 88.9 | 7.2 | 88.8 | 9.5 | 5 |
|  | Musical | 49.1 | 6.5 | 63.8 | 10.9 | 31 |
|  | Intelligibility | 35.2 | 8.8 | 58.8 | 9.7 | 87 |
|  | Combines words | 27.8 | 1.7 | 47.5 | 9.8 | 76 |
| 9y | CCC – intelligibility & fluency | 75.9 | 11.1 | 75.0 | 9.6 | 20 |
|  | CCC – syntax score | 78.2 | 11.1 | 77.5 | 9.6 | 16 |
|  | CCC – coherence | 96.4 | 9.7 | 91.3 | 9.2 | 2 |
| 42m | Rutter Prosocial | 72.7 | 8.8 | 67.5 | 11.6 | 12 |
| 47m | SDQ Prosocial | 68.3 | 8.6 | 61.3 | 7.5 | 17 |
| 57m | Empathy | 58.9 | 9.9 | 66.3 | 8.5 | 14 |
| 69m | Empathy | 71.9 | 12.9 | 66.3 | 9.6 | 9 |
| 81m | Empathy | 70.9 | 13.5 | 66.3 | 9.6 | 11 |
|  | SDQ Prosocial | 62.5 | 9.2 | 58.8 | 7.2 | 18 |
| 91m | SCDC | 87.5 | 9.3 | 78.8 | 9.6 | 6 |
| 97m | SDQ Prosocial | 72.9 | 12.9 | 63.8 | 9.7 | 19 |
| 9y | SDQ Prosocial | 63.6 | 7.2 | 62.5 | 9.9 | 22 |
|  | CCC – conversational rapport | 83.6 | 7.5 | 83.8 | 9.5 | 4 |
| 38m | Echoes what said | 38.1 | 21.4 | 30.0 | 15.8 | 99 |
| 57m | Echoes what said | 15.3 | 2.1 | 13.8 | 2.0 | 69 |
| 69m | Echoes what said | 12.5 | 1.1 | 48.8 | 14.8 | 67 |
| 81m | Echoes what said | 63.3 | 16.8 | 43.8 | 10.4 | 61 |
|  | Nonverbal communication | 42.1 | 5.0 | 62.5 | 12.5 | 38 |
| 8y | WOLD – comprehension | 44.4 | 13.1 | 57.5 | 9.1 | 45 |
|  | WOLD – oral expression | 33.3 | 6.8 | 60.0 | 9.7 | 27 |
|  | Nonword repetition | 40.0 | 8.0 | 52.5 | 9.9 | 42 |
|  | WISC – verbal IQ | 47.6 | 10.4 | 73.8 | 9.6 | 13 |
|  | DANVA – faces | 29.6 | 9.2 | 55.0 | 8.7 | 56 |
| 9y | CCC – inappropriate initiation | 52.7 | 10.2 | 45.0 | 11.1 | 60 |
|  | CCC – stereotyped conversation | 64.8 | 10.0 | 57.5 | 11.1 | 29 |
|  | CCC – conversational context | 85.5 | 7.1 | 83.8 | 9.5 | 3 |
| 18m | Repetitive behaviour | 25.7 | 12.6 | 25.0 | 10.7 | 93 |
| 30m | Repetitive behaviour | 30.0 | 5.5 | 33.8 | 9.8 | 78 |
| 42m | Repetitive behaviour | 38.5 | 5.5 | 45.0 | 9.8 | 66 |
| 57m | Repetitive behaviour | 45.2 | 5.5 | 50.0 | 9.7 | 48 |
| 69m | Repetitive behaviour | 61.0 | 6.7 | 62.5 | 9.7 | 40 |
| 77m | Repetitive behaviour | 51.7 | 5.3 | 57.5 | 9.7 | 47 |
| 91m | DAWBA – Number compulsions | 68.5 | 9.1 | 72.5 | 9.6 | 26 |
|  | DAWBA – Compulsions score | 68.5 | 9.1 | 70.0 | 9.6 | 23 |
|  | DAWBA – Tics or twitches | 11.1 | 2.0 | 32.5 | 9.9 | 94 |
| 38m | Stumbles on words | 13.1 | 5.7 | 10.0 | 4.8 | 101 |
|  | Prefers gestures | 63.6 | 8.8 | 61.3 | 9.7 | 41 |
| 57m | Stumbles on words | 20.7 | 3.0 | 22.5 | 2.7 | 88 |
|  | Prefers gestures | 39.7 | 3.3 | 42.5 | 3.5 | 52 |
|  | Pronouncing certain sounds | 62.1 | 28.2 | 47.5 | 20.2 | 89 |
| 69m | Stumbles on words | 29.8 | 1.7 | 48.8 | 16.2 | 68 |
|  | Prefers gestures | 33.9 | 2.6 | 33.8 | 2.7 | 53 |
|  | Pronouncing certain sounds | 60.7 | 20.0 | 43.8 | 13.1 | 82 |
| 81m | Stumbles on words | 60.4 | 17.4 | 51.3 | 11.5 | 49 |
|  | Prefers gestures | 28.6 | 1.7 | 30.0 | 1.8 | 34 |
|  | Pronouncing certain sounds | 50.0 | 13.3 | 46.3 | 9.8 | 77 |
| 38m | EAS – Sociability | 46.3 | 11.2 | 43.8 | 8.9 | 55 |
|  | Stays mainly silent | 52.5 | 8.9 | 57.5 | 9.7 | 51 |
|  | Avoids eye contact | 58.1 | 17.5 | 50.0 | 13.0 | 64 |
| 57m | EAS – Sociability | 43.5 | 6.1 | 50.0 | 10.2 | 46 |
|  | Stays mainly silent | 48.2 | 17.2 | 40.0 | 11.8 | 80 |
|  | Avoids eye contact | 12.3 | 1.8 | 58.8 | 17.0 | 65 |
| 69m | EAS – Sociability | 50.8 | 5.9 | 58.8 | 9.7 | 28 |
|  | Stays mainly silent | 6.9 | 0.2 | 35.0 | 12.4 | 84 |
|  | Avoids eye contact | 12.7 | 2.0 | 61.3 | 15.6 | 62 |
| 81m | Stays mainly silent | 8.5 | 0.1 | 26.3 | 12.5 | 85 |
|  | Avoids eye contact | 23.5 | 1.5 | 60.0 | 14.0 | 43 |
| 91m | DAWBA – Social fears | 28.6 | 7.8 | 45.0 | 9.2 | 72 |

For a list of abbreviations associated with the individual measures see Methods S3. *Observed data* excludes imputed values (N = 6820 to 11346). *All data* includes observed and imputed values (N=13138). With few ASD cases, the prevalence of the worst decile for each trait in non-ASD children, equivalent to (1 – specificity), was close to the actual 10% cut-off used. Deviations from the nominal value reflected clustering on the categorical responses. Results for the factors are not shown for *Observed data* since this related to 2481 observations with only four ASD cases. The 93 individual measures, the 7 factors and the Factor mean score are ranked in terms of their explanatory power of ASD after adjusting for gender using *All data* assuming linearity of the trait. The number of ASD cases varied by age and trait for *Observed data*: 68 (6m), 74 (15m & 18m), 69 – 70 (24m), 68 – 70 (30m), 61 – 67 (38m), 65 – 66 (42m), 63 (47m), 56 – 63 (57m), 55 – 60 (69m), 58 (77m), 47 – 57 (81m), 49 – 56 (91m), 48 (97m), 21 – 27 (8y) and 54 – 55 (9y). There were 80 ASD cases for *All data*.
