# Supplementary material for: Traits Contributing to the Autistic Spectrum
Source: PLoS One. 2010 Sep 8;5(9):e12633. doi: 10.1371/journal.pone.0012633 (PMC2935882; doi:10.1371/journal.pone.0012633)
Supplement: Table S7 — Subset regression results of 93 individual measures on ASD status adjusting for gender. (0.05 MB DOC) [file pone.0012633.s011.doc]

Table S7: Subset regression results of 93 individual measures on ASD status adjusting for gender

|  | Subset size 3 | | | | | | | | Subset size 4 | | | | | |
| --- | --- | --- | --- | --- | --- | --- | --- | --- | --- | --- | --- | --- | --- | --- |
|  | Unrestricted models | | | | Restricted models | | | | Unrestricted models | | | Restricted models | | |
| CCC – coherence 9y | X |  | X | X | X | X | X | X | X | X |  |  | X | **X** |
| CCC – conversational context 9y |  | X |  |  |  |  |  |  |  | X | X | X |  |  |
| Rutter Prosocial 42m |  | X | X |  |  |  |  |  | X |  | X | X | X |  |
| SCDC 91m | X |  |  | X |  |  |  | X | X | X | X |  |  | **X** |
| EAS – Sociability 38m |  |  |  | X |  |  | X |  |  |  |  |  |  | **X** |
| EAS – Sociability 69m | X | X | X |  | X | X |  |  | X | X | X | X | X |  |
| Repetitive behaviour 69m |  |  |  |  | X |  | X | X |  |  |  | X | X | **X** |
| Repetitive behaviour 77m |  |  |  |  |  | X |  |  |  |  |  |  |  |  |
| Increase in R^2^ (N=13138) | 0.4450 | 0.4397 | 0.4382 | 0.4354 | 0.4221 | 0.4161 | 0.4130 | 0.4124 | 0.4612 | 0.4509 | 0.4504 | 0.4456 | 0.4446 | **0.4407** |
| N (at least one observed) | 10248 | 11163 | 11185 | 11225 | 9884 | 10248 | 11258 | 10242 | 11330 | 10250 | 11313 | 11169 | 11190 | **11411** |

For a list of abbreviations associated with the individual measures see Methods S3.

Unrestricted models selected the best individual measures from any of the diagnostic triads. Restricted models contained at least one measure from each of the triads. Only the best four models are shown for subset size 3 and the best 3 models for subset size 4. Increase in R^2^ reflected the increase in the explanation of the log likelihood compared to a model involving gender only. For comparative purposes, all models used the same number of observations although the balance between observed and imputed data varied between models. The number of observations is reported where at least one measure in the model was observed. X indicates inclusion in the model.

Examination of the results for size 3 suggested that duplicate measures in the social domain could significantly add to the explanation of ASD. Consequently, analyses were repeated for size 4. Of the restricted models, the one shown in bold was selected although inferior to other models on the R^2^ criterion. The basis for selection was that the marginal increase in N (2%) was more important than the marginal decrease in R^2^ (1%).

Although there was further evidence that 3 duplicate measures of the social domain or two duplicate measures of the communication domain could significantly add to the explanation of ASD, the R^2^ for size 5 improved by <0.009 compared to the best restricted model of size 4.
