# Supplementary figures and images for: Traits Contributing to the Autistic Spectrum
Source: PLoS One. 2010 Sep 8;5(9):e12633. doi: 10.1371/journal.pone.0012633 (PMC2935882; doi:10.1371/journal.pone.0012633)

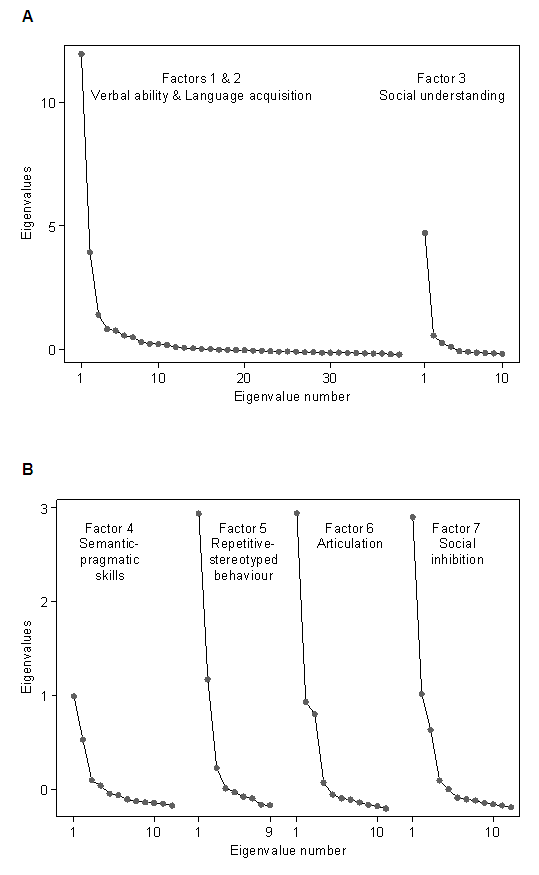

Supplement: Figure S1 — Scree plots from factor analyses of individual measures associated with each factor. While a combined analysis of all 93 measures has identified the major factors, there was evidence that more minor factors existed in a hierarchical structure (see Table 1, Figure 1). These minor factors may be more apparent in separate analyses of measures associated with a single factor rather than in combined analyses. In part A, the factor structure of measures associated with factors 1 to 3 was not further differentiated. But analysis of measures associated with factors 4 to 7 in part B, showed the possibility of more minor factors. The factor structure became differentiated with duplicate measures clustering on the same factor. The definition of ‘duplicate’ varied between factors. Hence for the analysis of Factor 4, the split was by questionnaire/clinic measures. For other factors, different questions formed different factors with repeat measures clustering on the same factor. In particular for Factor 5, DAWBA measures clustered on the same factor. These four major factors might be separated into 10 minor factors. (0.03 MB TIF) [file pone.0012633.s012.tif]

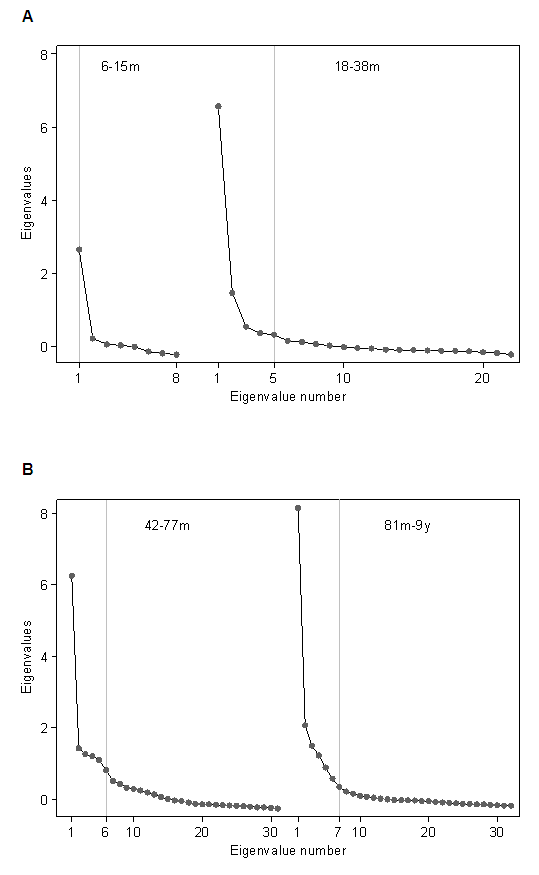

Supplement: Figure S2 — Scree plots from factor analyses of individual measures relating to different age ranges. Analysis of 8 traits at 6–15 m and 22 traits at 18–38 m (part A) and, 31 traits at 42–77 m and 32 traits at 81 m–9y (part B) suggested 1, 5, 6 and 7 factors respectively. (0.03 MB TIF) [file pone.0012633.s013.tif]
